# Atlantic herring (*Clupea harengus*) population structure in the Northeast Atlantic Ocean

**DOI:** 10.1016/j.fishres.2022.106231

**Published:** 2022-05

**Authors:** Sunnvør í Kongsstovu, Svein-Ole Mikalsen, Eydna í Homrum, Jan Arge Jacobsen, Thomas D. Als, Hannes Gislason, Paul Flicek, Einar Eg Nielsen, Hans Atli Dahl

**Affiliations:** a Amplexa Genetics A/S, Hoyvíksvegur 51, FO-100 Tórshavn, Faroe Islands; b University of the Faroe Islands, Faculty of Science and Technology, Vestara Bryggja 15, FO-100 Tórshavn, Faroe Islands; c Faroe Marine Research Institute, Nóatún 1, FO-100 Tórshavn, Faroe Islands; d Aarhus University, Department of Biomedicine, Høegh-Guldbergs Gade 10, 8000 Aarhus C, Denmark; e European Molecular Biology Laboratory, European Bioinformatics Institute, Wellcome Genome Campus, Hinxton, Cambridge CB10 1SD, UK; f DTU Aqua – National Institute of Aquatic Resources, Technical University of Denmark, Vejlsøvej 39, 8600 Silkeborg, Denmark

**Keywords:** Atlantic herring, Low coverage sequencing, Population structure, Stock assignment

## Abstract

The Atlantic herring *Clupea harengus* L has a vast geographical distribution and a complex population structure with a few very large migratory units and many small local populations. Each population has its own spawning ground and/or time, thereby maintaining their genetic integrity. Several herring populations migrate between common feeding grounds and over-wintering areas resulting in frequent mixing of populations. Thus, many herring fisheries are based on mixed populations of different demographic status. In order to avoid over-exploitation of weak populations and to conserve biodiversity, understanding the population structure and population mixing is important for maintaining biologically sustainable herring fisheries. The aim of this study was to investigate the genetic population structure of herring in the Faroese and surrounding waters, and to develop genetic markers for distinguishing between four herring management units (often called stocks), namely the Norwegian spring-spawning herring (NSSH), Icelandic summer-spawning herring (ISSH), North Sea autumn-spawning herring (NSAH), and Faroese autumn-spawning herring (FASH). Herring from the four stocks were sequenced at low coverage, and single nucleotide polymorphisms (SNPs) were called and used for population structure analysis and individual assignment. An ancestry-informative SNP panel with 118 SNPs was developed and tested on 240 individuals. The results showed that all four stocks appeared to be genetically differentiated populations, but at lower levels of differentiation between FASH and ISSH than the other two populations. Overall assignment rate with the SNP panel was 80.7%, and agreement between the genetic and traditional visual assignment was 75.5%. The NSAH and NSSH samples had the highest assignment rate (100% and 98.3%, respectively) and highest agreement between traditional and genetic assignment methods (96.6% and 94.9%, respectively). The FASH and ISSH samples had substantially lower assignment rates (72.9% and 51.7%, respectively) and agreement between traditional and genetic methods (39.5% and 48.4%, respectively)

## 1 Introduction

Atlantic herring (*Clupea harengus* L.) is an important economical and nutritional resource for the countries around the North Atlantic, as well as an important part of the North Atlantic marine ecosystem (Holst et al., 2004). Therefore, it is crucial to keep the fisheries sustainable. Herring fisheries are managed as units, referred to as stocks, which represents the geographical unit of assessment and management, which in some instances are equal to putative genetic populations, but commonly are not (McPherson et al., 2001). Smith et al. (1991) showed how neglecting to account for population structure in fisheries management can result in overexploitation and the loss of genetic diversity. Therefore, knowing whether a stock represent a true biological population is of great importance to ensure that fisheries target the intended population, as well as to set realistic regulations for fisheries management. Moreover, elucidation of population structure is crucial for understanding the distributional range and migration behaviour of the species. Thus, identification of population structure is important for maintaining sustainable fisheries, and can also be used as a genetic tool in the fight against illegal, unreported, and unregulated fishing (Dahle et al., 2018; Nielsen et al., 2012), as well as in the forensic identification of fish and fish products throughout the food processing chain (Nielsen, 2016).

Numerous herring populations exist in the Northeast Atlantic, each with their own spawning ground and/or time. Herring population structure is complex with a few very large migratory units and many small local populations (Hay et al., 2001). For example, the herring stocks both within the Baltic Sea and around the British Isles consists of several populations (Jørgensen et al., 2005; Ruzzante et al., 2006). This means that herring fisheries often consist to some degree of mixed catches and distinguishing between the different populations can be problematic. Morphological, physiological, and biological characteristics are traditional parameters used for assigning individuals back to a population, but these characteristics are subject to interpretation (Pampoulie et al., 2015). Examples of applied phenotypic methods for distinguishing between herring populations include vertebrate count, otolith outline, and otolith microchemistry (Geffen et al., 2011; Hulme, 1995; Libungan et al., 2015). These methods have been able to distinguish between herring populations to a varying degree, but a downside of these characteristics is that they are affected by the environment and therefore serve merely as proxies for genetic differences. Different genetic methods based on microsatellites and/or single nucleotide polymorphisms (SNPs) have also been applied in order to investigate the population structure of Atlantic herring. McPherson et al. (2004) demonstrated a significant difference between Atlantic herring in the Northeast and Northwest Atlantic, as well as among spawning groups in the Northwest Atlantic. Other studies have shown that both the Baltic herring and North Sea herring are genetically distinct from other herring populations in the Northeast Atlantic (Lamichhaney et al., 2012; Limborg et al., 2012). Furthermore, distinct populations have been found within the Baltic Sea (Bekkevold et al., 2016; Corander et al., 2013; Teacher et al., 2013).

In Faroese waters, herring population structure and mixing is partially unknown. The local Faroese autumn-spawning herring (FASH), also called the fjord herring, is the only population assumed to currently spawn in Faroese waters. This population occurs sporadically, and the exact spawning locations are unknown. Nevertheless, this fjord herring has been observed intermittently since at least the 1780s (Joensen and Tåning, 1970). During parts of the year, the Norwegian spring-spawning herring (NSSH), which historically also spawned in Faroese waters (Joensen and Tåning, 1970; Tåning, 1943), the North Sea autumn-spawning herring (NSAH) (Holst et al., 2004; Jacobsen, 1991) and the Icelandic summer-spawning herring (ISSH) (Jakobsson et al., 1969) are assumed to mix with FASH to some degree in the areas where Faroese waters meet Icelandic, British and Norwegian waters. Consequently, the Faroese herring fishery can be considered to be mixed-stock fishery. Morphological features have therefore been examined from Faroese catches to assign the herring individuals back to stocks. Few genetic studies have been carried out that included samples from the FASH population and they have not been able to distinguish it from the other Northeast Atlantic herring (Bekkevold et al., 2015; Pampoulie et al., 2015; Skírnisdóttir et al., 2012). Likewise, only one study (Shaw et al., 1999) has shown genetic differentiation between ISSH and NSSH, whereas others have not been able to replicate this (Pampoulie et al., 2015). Accordingly, studies with higher resolution are needed in order to resolve the herring population structure in Faroese waters.

Recent studies of the herring genome have revealed that genomic differentiation between populations is generally low, but that there are specific regions in the genome that show elevated genetic differentiation associated with ecological adaption to e.g., spawning time and salinity (Han et al., 2020; Pettersson et al., 2019). Thus, scanning the entire genome for regions of high differentiation is expected to be a more powerful method for population identification and for developing panels for fast population assignment in relation to fisheries management. Therefore, the overarching aim of this study was to provide a case study of the application of low coverage genome sequencing to resolve the population structure of high gene flow marine fish populations with presumed shallow evolutionary histories influenced by adaptive evolution. The focus was to investigate population structure and mixing of herring in Faroese waters and specifically investigate whether the FASH stock is a genetically distinct population. A second aim was to mine the genome for genetic markers that could distinguish between the four herring stocks in the Northeast Atlantic and to apply them for individual assignment in relation to fisheries management.

## 2 Materials and methods

### 2.1 Sample collection

**Fig. 1:**
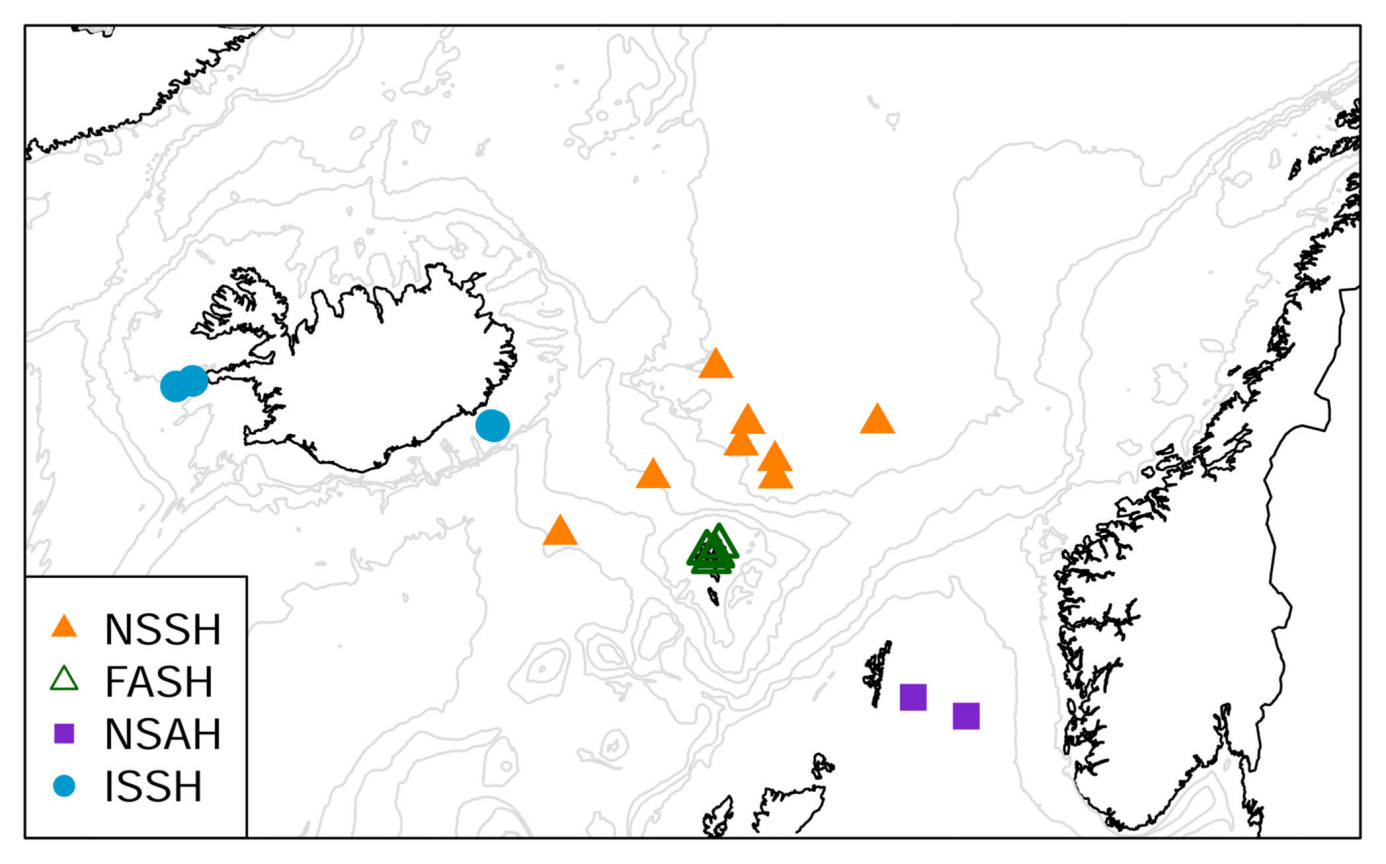
Sampling sites of the Atlantic herring used in this study. Assumed populations are: NSSH = Norwegian spring-spawning herring (▴), NSAH = North Sea autumn-spawning herring (■), FASH = Faroese autumn-spawning herring (Δ), and ISSH = Icelandic summer-spawning herring (•).

Herring samples were obtained from several sources. They were collected on a research cruise conducted by the Faroe Marine Research Institute (FAMRI), obtained from fishing boats, or kindly provided by NAFC Marine Centre in Scalloway and the Marine Research Institute in Iceland. The length, weight, sex, and maturity stage of the fish were recorded, and the otoliths extracted. The maturity stage was classified according to the local maturity scale which is based on Bowers and Holliday (1961). This maturity scale and a conversion to the WKASMSF (Workshop for Advancing Sexual Maturity Staging in Fish) 2018 scale (ICES, 2018) is provided in Supplementary Table A1 in Appendix A. The fish age and spawning type were inferred from the otoliths; a hyaline otolith nucleus indicated autumn spawners and an opaque nucleus indicated spring spawners (Postuma, 1974). The maturity stage and spawning type (autumn/spring) were used, together with the location and time of catch, to identify which stock the individual fish belonged to. Hereinafter this will be referred to as the traditional assignment method. These are the methods used by FAMRI to assign herring from fisheries to stocks. These stocks are assumed to be biological populations and will hereinafter be referred to as populations. The sampling sites are shown in Fig. 1, and Supplementary Table A2 (Appendix A) shows the time of catch and maturity stage distribution of the samples.

### 2.2 DNA extraction and sequencing

Due to the uncertainties surrounding the FASH exact spawning place and time, collecting only spawning individuals for the baseline was not possible. Therefore, the genetic populations for the baseline were defined in a two-step process. First the individuals for sequencing were chosen based on traditional assignment (see Section 2.1). This was done to ensure a roughly even representation of the four putative populations in the sequenced samples. These samples were subjected to low coverage whole genome sequencing. The second step was to define the baseline populations using genetic methods (see Section 2.5).

The DNA was extracted from tissue samples from 103 herring (29 NSSH, 30 ISSH, 17 NSAH, and 27 FASH [traditional assignment method]) using an AS1000 Maxwell 16 instrument (Promega, Wisconsin, United States) and the Maxwell 16 Tissue DNA Purification Kit (Promega). DNA concentration was measured using a Qubit 3.0 fluorometer (ThermoFisher Scientific, Massachusetts, United States). The DNA was fragmented to roughly 300 bp using a Covaris M220 (Covaris, Chicago, United States) and the libraries were prepared using the KAPA LTP Library Preparation Kit for Illumina Platforms (KAPABiosystems, Massachusetts, United States), following the manufacturer’s instructions. The library from each individual herring was indexed using indexed adapters (Pentabase, Odense, Denmark) and quantified using the KAPA Library Quantification Kit (KAPABiosystems), following the manufacturer’s instructions. After quantification, the libraries were pooled to equal proportions and sequenced on a NextSeq500 (Illumina, California, United States) using the High Output v2 Kit (Illumina).

### 2.3 Sequencing data pre-processing and SNP calling

The sequencing data were trimmed to remove adapters and low-quality bases (Q score < 20) using Trimmomatic v0.36 (Bolger et al., 2014). AfterQC v0.4.0 was used to remove the polyG reads (Chen et al., 2017), and FastQC v0.11.5 was used to assess the quality of all the sequencing data (Andrews, 2010). The data were then aligned to the draft herring genome (GCF_000966335.1_ASM96633v1) using BWA-MEM v0.7.15 (Li, 2013). Furthermore, SAMtools v1.3 was used for sorting, converting, and removing PCR duplicates from the alignment files (Li et al., 2009). SNPs were called using FreeBayes v1.1.0 (Garrison and Marth, 2012), with pooled data (sequencing reads from individuals from the same population were pooled) and individual data. Low-quality SNPs (QUAL < 20) were filtered out from both the individual and pooled data sets. For the individual data set, SNPs where fewer than 30 individuals had data (number of samples with data [NS] < 30) were also filtered out, and for the pooled data set, SNPs with NS < 4 were filtered out. Genotype likelihoods were also called from the BAM files using ANGSD, with the p-value cut-off set at 10^-6^. SNPs where more than 90 individuals had missing genotype likelihood as well as those with a minor allele frequency lower than 0.05 were filtered out.

### 2.4 SNP panel

SNP-wise Weir and Cockerham F_ST_ (Weir and Cockerham, 1984) were calculated for pairwise combinations of putative populations (determined via traditional methods) using VCFtools v0.1.15 (Danecek et al., 2011). For every pairwise comparison, the 100 SNPs with the highest F_ST_ were selected. The duplicates were removed and the genotypes for these SNPs were extracted from the individual sequencing data. Plink v1.07 (Purcell et al., 2007) was then used to calculate the linkage disequilibrium (LD) and to prune the SNPs based on the LD. The 154 remaining SNPs were used for further analysis. After genotyping of the assessment individuals (see Section 2.6) 118 SNPs were successfully genotyped and were used in the final SNP panel (Supplementary Table A3 in Appendix A lists these SNPs).

### 2.5 Population structure of baseline samples

The genetic population structure of the baseline samples was investigated with a principal component analysis (PCA) using the genotype likelihoods from the full SNP data set and PCAngsd from the ANGSD R package (Meisner and Albrechtsen, 2018; Korneliussen et al., 2014), which uses the genotype likelihoods and works well with low sequencing coverage (Skotte et al., 2013). The population structure was further investigated with a discriminant analysis of principal components (DAPC) using the SNP panel genotypes and the Adegenet R package (Jombart and Ahmed, 2011). The find.clusters function was used to define the populations (hereinafter referred to as the baseline populations), which were used as the reference populations in the subsequent analyses. To validate the population structure, the most likely number of populations (K) was calculated using the SNP panel genotypes and STRUCTURE v2.3.4 (Falush et al., 2003), with an admixture model with correlated allele frequencies but without information on sample location. Ten independent STRUCTURE runs for K = 1–8 with 100,000 burn-ins and 100,000 iterations were carried out. Subsequently, Clumpak (Kopelman et al., 2015) was used to estimate the optimal number of K according to the Evanno method (Evanno et al., 2005). The Adegenet R package was also used to perform a DAPC with the baseline samples and SNP panel data set. Lastly, pairwise population F_ST_ values for the SNP panel data set were calculated using the Genepop R package (Rousset, 2008) and for the full SNP data set using VCFtools v0.1.15. A population assignment test using Monte-Carlo cross--validation was done using the R package assignPOP (Chen et al., 2018) and the 118 SNPs from the final SNP panel (Section 2.4). Ninety iterations were run with all loci and 50% of the individuals were used for training.

### 2.6 Assessment samples - Genotyping

Tissue samples from 240 herring (60 from each putative population [based on traditional assignment]) were sent to LGC Genomics (Berlin, Germany) for genotyping, and 500 predetermined SNPs were genotyped using their SeqSNP service. These 500 SNPs were selected based on their discriminatory power between the populations; 154 SNPs were selected based on the pooled data (Section 2.4); and the rest were selected based on the individual-level data (using the same method). Twenty-eight technical replicas (duplets) were included. The DNA extracted from these tissue samples was highly fragmented (fragment sizes below 1 kb) because several of our samples were from fisheries catches and had been frozen and thawed twice before the DNA was extracted. The LGC SeqSNP method is best suited for fragments of 10 kb and higher. Nevertheless, these samples were typical samples from fishery landings, the type of samples in need of individual assignment in the event of mixed fisheries. For this reason, we chose to perform the genotyping experiment with the low-quality DNA.

### 2.7 Assessment samples - Assignment

We investigated the agreement between the traditional assignment method and genetic assignment methods. The assessment samples were assigned to populations using the traditional assignment method described in Section 2.1. The genetic assignment was carried out using the SNP panel genotyping information with the three following methods: 1) The assign.X function from the assignPOP R package with default parameters and the baseline populations as reference populations. Assignment in assignPOP is based on Monte-Carlo and K-fold cross-validation procedures, as well as PCA analysis (Chen et al., 2018). 2) The predict.dapc function from the Adegenet R package with the DAPC from the baseline samples as reference. 3) The GeneClass2 software with options: Assign/Exclude population as origin of individuals and the Rannala & Mountain Bayesian method. Individuals where only classified as assigned if all three genetic methods agreed, otherwise they were classified as unassigned.

## 3 Results

### 3.1 Sequencing, SNP calling, and genotyping

Sequencing of the 103 baseline herring genomes resulted in a total coverage of 267x and an average coverage of 2.6x at the individual level. Table 1 presents the coverage for each putative population (based on traditional assignment). After filtering, 4,902,721 SNPs from the individual data were kept (referred to as the full SNP data set), while 8,737,355 SNPs from the pooled data were kept. A panel of SNPs was selected based on pairwise F_ST_ values (see Section 2.4) and further narrowed based on LD, leading to a selection of 154 SNPs with the highest discriminatory power.

Genotyping of 240 individuals (assessment samples) for 500 SNPs were performed and after filtering 377 SNPs and 238 individual herring remained (see Section 2.6). Of these 377 SNPs, 118 overlapped with the original 154 selected SNPs, resulting in a final panel of 118 SNPs (hereinafter referred to as the SNP panel).

### 3.2 Population structure

The genetic baseline populations were defined using the 103 baseline individuals, the SNP panel data set and a DAPC analysis. The individuals constituting the baseline populations can be found in the Supplementary Table A4 (Appendix A). The population clusters identified generally corresponded well with the traditional population identification of the individuals, but a number of individuals were identified among the baseline that were assigned to the wrong population by traditional methods. For example, five individuals supposedly from the FASH population (traditional methods), were shown to belong to the NSAH population by genetic analysis, thus indicating some population mixing in the Faroese fjords. A population structure analysis with the full SNP data set was also carried out using the ANGSD R package. The results from this analysis showed the same clustering pattern as the DAPC results (Supplementary Figure A1 in Appendix B).

**Fig. 2:**
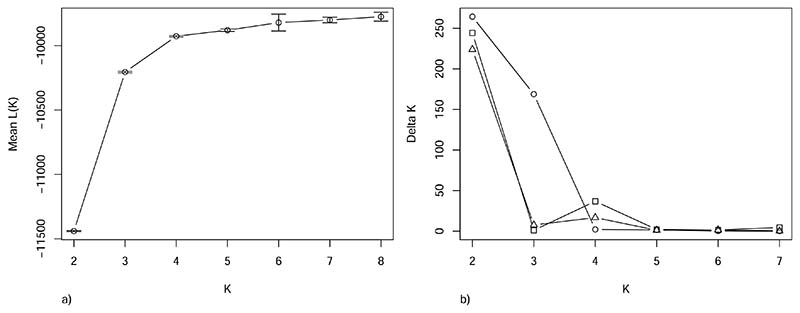
Mean likelihood of K and delta K from the STRUCTURE analyses. a) Mean likelihood of K ± standard deviation given data from the STRUCTURE analyses using all four baseline populations: Norwegian spring-spawning herring (NSSH), North Sea autumn-spawning herring (NSAH), Faroese autumn-spawning herring (FASH), and Icelandic summer-spawning herring (ISSH). b) Delta K from the Evanno method for determining the most likely number of K, using STRUCTURE results. The data points show the number of populations used in the analysis: = NSSH, NSAH, FASH, and ISSH. □ = NSAH, FASH, and ISSH. Δ = FASH and ISSH. All analyses were run with an admixture model with correlated allele frequencies, but without information on sample location for 10 independent runs for K = 1–8 with 100,000 burn-ins and 100,000 iterations.

**Fig. 3:**
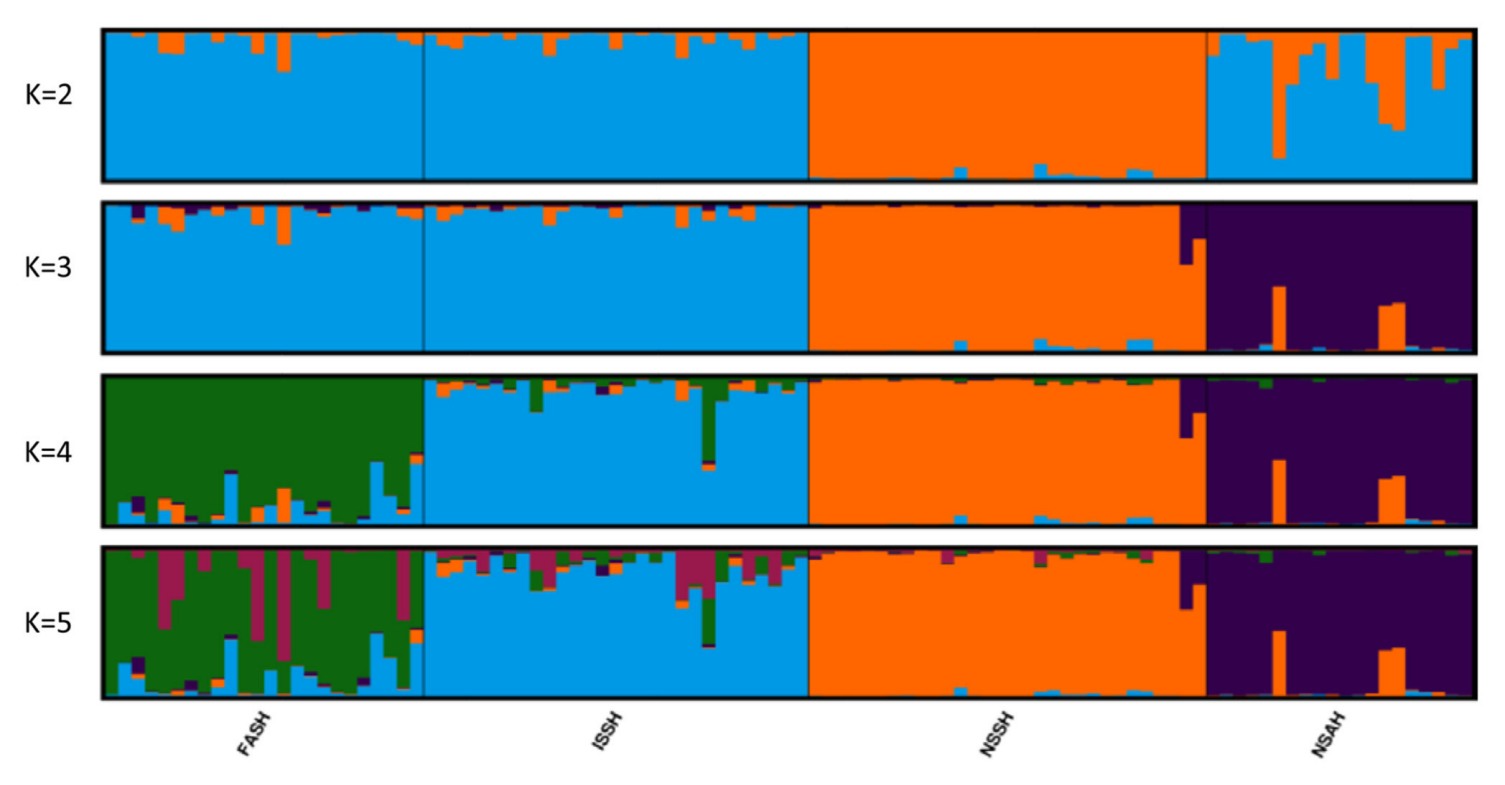
Barplots of individual admixture proportions from the STRUCTURE analysis showing Atlantic herring population structure based on the genotype of 118 SNPs for K = 2–5. NSSH = Norwegian spring-spawning herring, NSAH = North Sea autumn-spawning herring, FASH = Faroese autumn-spawning herring, and ISSH = Icelandic summer-spawning herring.

The STRUCTURE analysis showed that the mean likelihood of K plateaued at K = 4 (Fig. 2a), indicating four populations, consistent with the baseline populations (Fig. 3). The Evanno method suggested two as the most likely number of clusters (Fig. 2b), with NSSH forming one cluster while the other three populations formed the second cluster (Fig. 3).

To search for substructures in the second cluster, the NSSH sample was removed, and the STRUCTURE and Evanno analyses were run again. This time the most likely number of K was again two (Fig. 2b) with the NSAH samples forming one cluster and ISSH and FASH the other (Supplementary Figure A3 in Appendix B). This indicated that there was also substructure in the second cluster. A third iteration, using only the ISSH and FASH samples, also provided K = 2. This indicated further substructure, confirming that there are most likely four populations in our baseline. These clusters correlate well with our baseline populations (Fig. 3). No further substructure was found. The results from the STRUCTURE analyses with K = 6–8 can be seen in the Supplementary Figure A2 (Appendix B), and for all Ks from the STRUCTURE analyses with data from three and two populations included in the analysis can be seen in Supplementary Figures A3 and A4 in Appendix B, respectively.

**Fig. 4:**
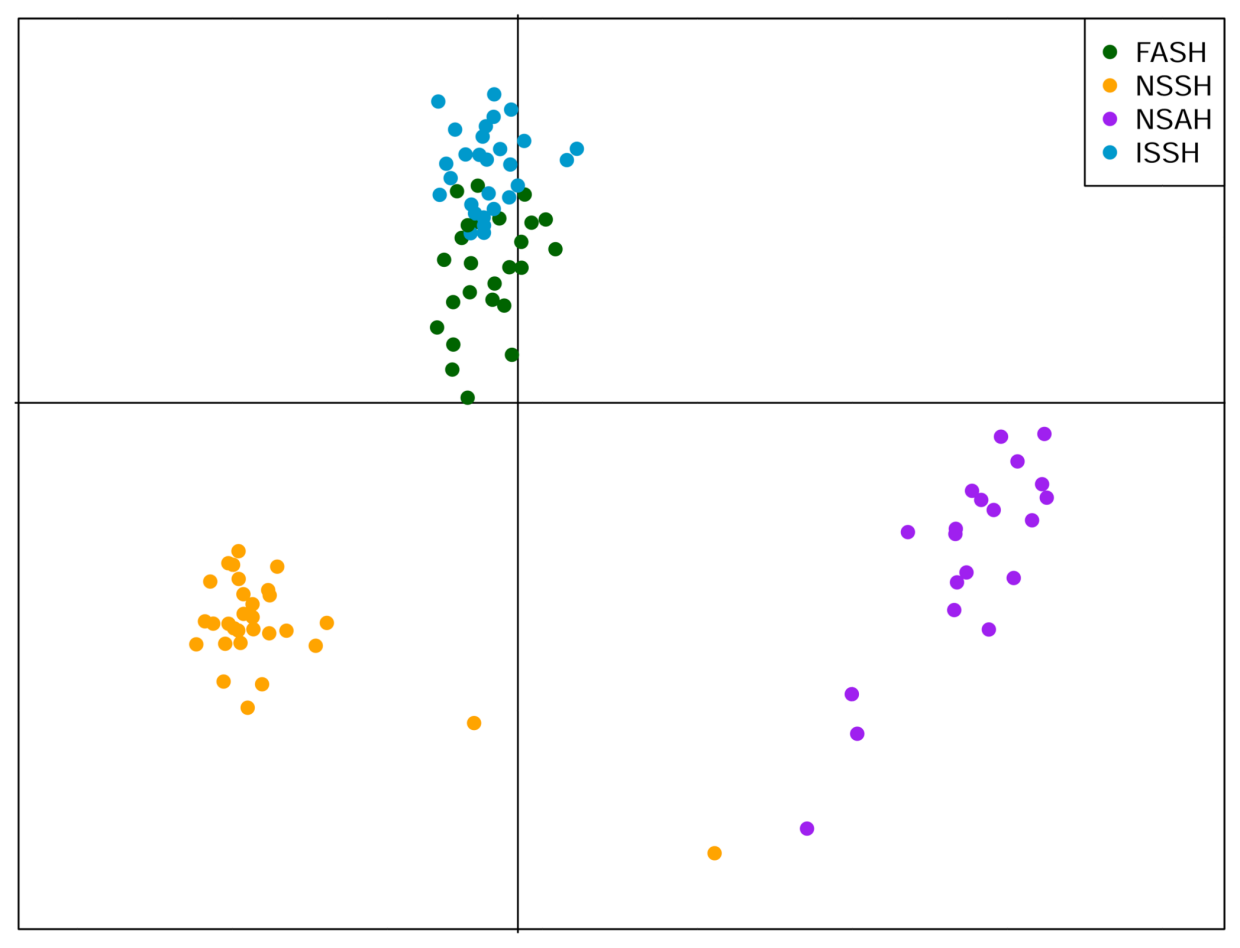
Discriminant analysis of principal components using baseline samples and the SNP panel. NSSH = Norwegian spring-spawning herring, NSAH = North Sea autumn-spawning herring, FASH = Faroese autumn-spawning herring, and ISSH = Icelandic summer-spawning herring.

The DAPC analysis of the SNP panel data set showed similar results as STRUCTURE. There were three distinct clusters (Fig. 4). The NSSH individuals clustered together, with the exception of two individuals. The NSAH individuals formed one loose cluster, suggesting more genetic variability and potentially additional substructure. The ISSH and FASH individuals clustered together, with the ISSH individuals almost exclusively at the top of the cluster and the FASH individuals at the bottom, with a slight overlap in the middle. This FASH-ISSH cluster was further inspected in a new DAPC containing only the FASH and ISSH samples which provided similar results as the first analysis (Supplementary Figure A5 in Appendix B).

The pairwise F_ST_ between the baseline populations was calculated using both the full data set and the SNP panel (Table 2). With the full data set, the F_ST_ values between the populations were low reflecting overall low genomic divergence but using the SNP panel, which was inflated with highly divergent regions of the genome, provided high levels of divergence and strong evidence for four different populations.

To test if the SNP panel could be used for assigning individuals to a population a test with the baseline samples and the R package assignPOP was performed. Ninety tests using Monte-Carlo cross-validation with 50% of individuals as training set were performed, to assign the rest of the individuals. The assignment accuracy was above 97% for all populations (Table 3). These results indicated that the SNP panel could be used to assign individuals to the populations.

### 3.3 Assessment samples

**Fig. 5:**
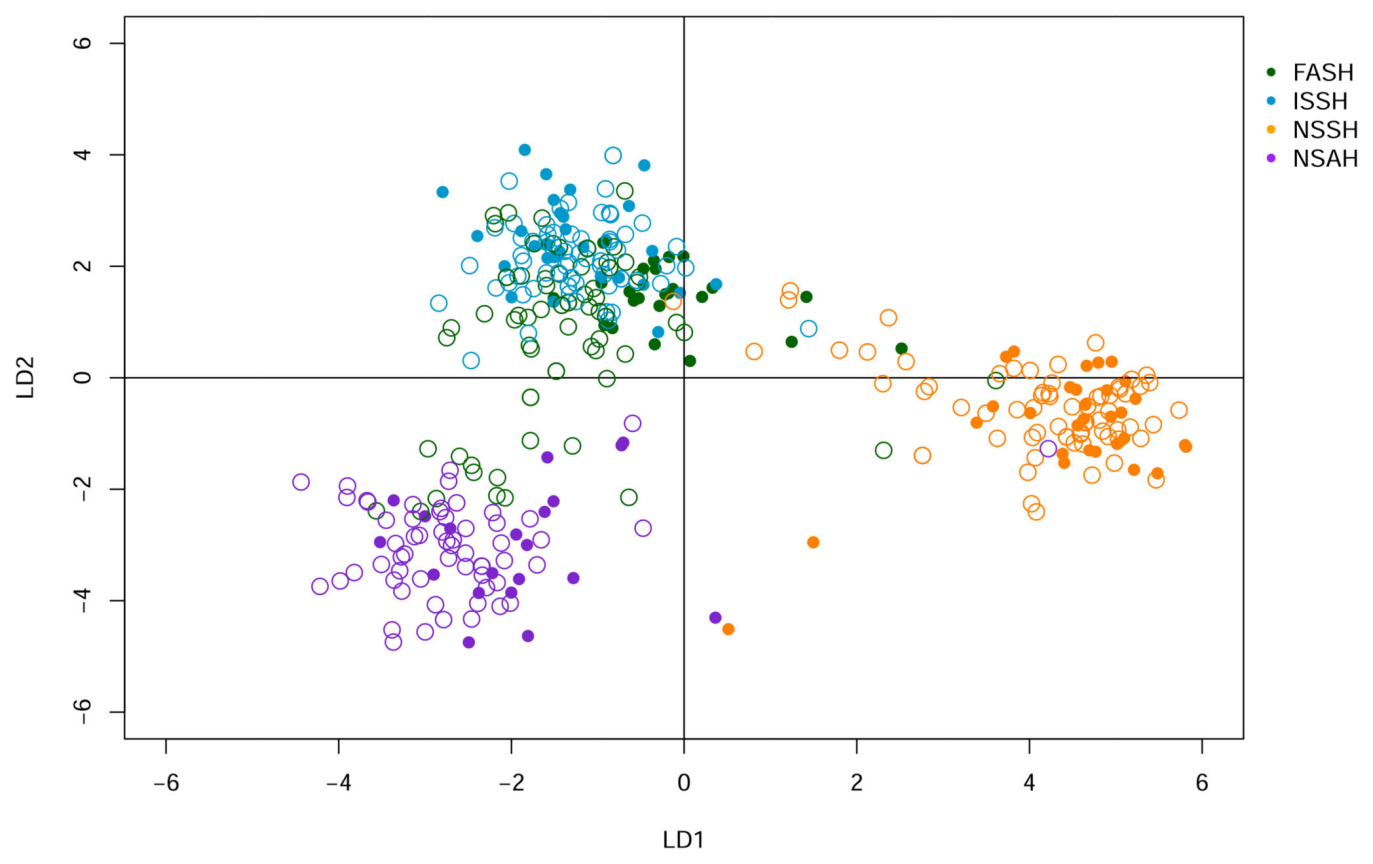
Discriminant analysis of principal components using baseline and assessment samples with SNP panel data. The filled circles represent the baseline samples, while the open circles represent the assessment samples. The colour of the circles indicates the population that the samples were assigned to using traditional methods. NSSH = Norwegian spring-spawning herring, NSAH = North Sea autumn-spawning herring, FASH = Faroese autumn-spawning herring, and ISSH = Icelandic summer-spawning herring.

The assessment individuals were assigned to a population using traditional visual methods and then genotyped for the 118 SNPs in the SNP panel. After filtering 238 individuals remained. A DAPC analysis with all 238 samples was performed to give an indication of the affinity between the assessment samples and the baseline samples. The assessment samples cluster in roughly the same pattern as the baseline samples (Fig. 5). It is evident that the traditional methods of assigning individuals to the populations provides some erronous assignments. There are especially many FASH individuals (determined by traditional methods) that according to the genotype data are more similar to the NSAH population.

The assessment samples were assigned to baseline populations in addition to the traditional population identification methods. Three different genetic algorithms were used to assign these assessment samples (GeneClass2 and the R packages AssignPOP and Adegenet). For 192 of the 238 individuals (80.7%) the assignment to a population was identical regardless of algorithm (Supplementary Table A6 in Appendix A). Out of these 192 unambiguously assigned individuals, 145 (75.5%) were assigned to the same populations using the traditional methods. The NSSH and NSAH samples have an assignment rate of above 98% and above 94% agreement between genetic and traditional assignment methods. The picture is quite different for FASH and ISSH; the assignment rate and agreement between methods for ISSH were 51.7% and 48.4%, respectively, for FASH the assignment rate and agreement between methods were 72.9% and 39.5%, respectively (Supplementary Table A6 in Appendix A).

**Fig. 6:**
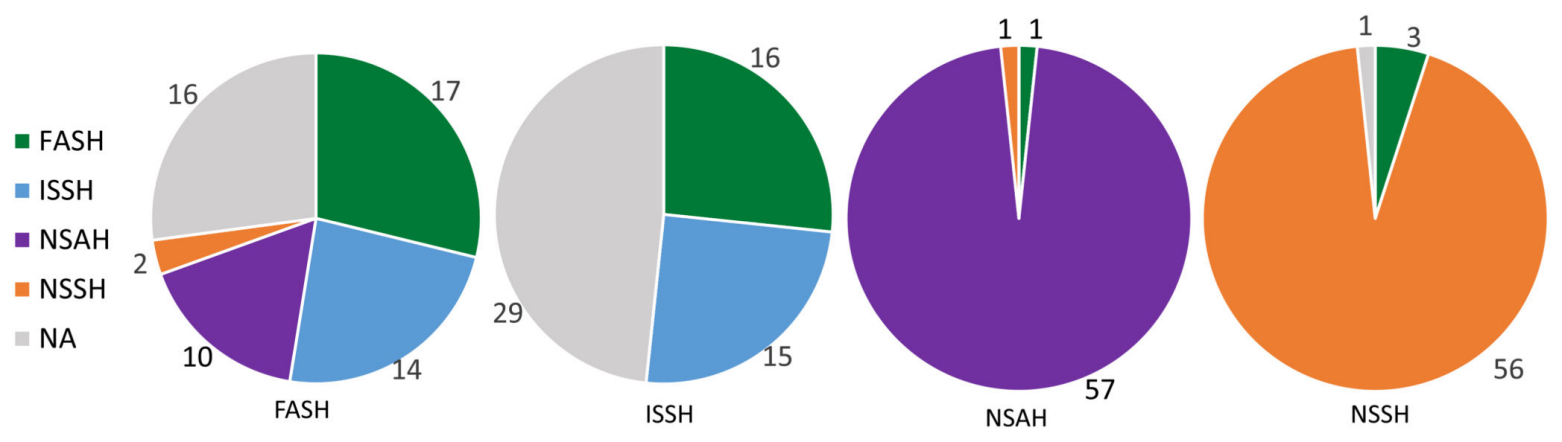
Traditional identification versus genetic assignment. The labels below the pies show which population of origin the assessment individuals were identified as belonging to using the traditional visual method, while the colours represent the genetic populations the same individuals were assigned to. NSSH = Norwegian spring-spawning herring, NSAH = North Sea autumn-spawning herring, FASH = Faroese autumn-spawning herring, and ISSH = Icelandic summer-spawning herring, NA = Not assigned.

Out of the 46 individuals not unambiguously assigned to a population, 43 pertained to problems with FASH or ISSH assignment (Fig. 6 and Supplementary Table A5 in Appendix A), likely caused by the lower level of genetic distinction between FASH and ISSH than the other two populations.

Twenty-five out of the 28 technical replicas resulted in identical assignment (Supplementary Table A5 in Appendix A). The three remaining replicates were all cases where the algorithms could not distinguish between ISSH and FASH, resulting in either the replicate being assigned to different populations, or one of them being unassigned.

Fig. 6 shows that the samples identified as belonging to FASH by traditional methods consists of several different genetic populations and that the genetic methods provide a higher resolution of the mixed population representing the local herring community. In addition, the ISSH sample appears to be a more mixed sample than expected; however, these individuals generally have the lowest assignment success rate.

### 3.4 Unexpected FASH individuals

The 13 spawning FASH individuals (traditional assignment; maturity stage 6 [actively spawning]) collected for this study, were captured from mid-August to mid-October (Supplementary Table A2 in Appendix A). The genetic analyses of these individuals gave unexpected results. Five were used in the baseline, of these three were determined as NSAH and two as FASH, after grouping with DAPC (Supplementary Table A4 in Appendix A). Eight spawning individuals were among the assessment samples, two of which were assigned to FASH, three to ISSH and three to NSAH (Supplementary Table A5 in Appendix A). Spawning NSAH have not been seen in Faroese waters before (see Section 4).

### 3.5 Chromosome regions that differ

**Fig. 7:**
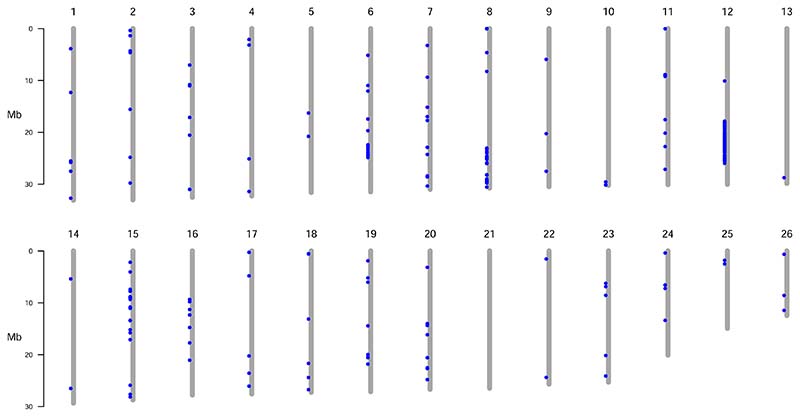
The chromosomal positions of the SNPs with the highest F_ST_ values between the four populations. The blue points represent the 457 high divergence SNPs identified from F_ST_ comparisons among the populations (see text for explanation).

As previously described, pairwise F_ST_ values for all SNPs between all population pairs were calculated and the top 100 SNPs from each pairwise comparisons selected. After removal of duplicates 457 SNPs remained (including the 118 panel SNPs). These SNPs were spread across the genome, with a few hotspots (Fig. 7). Half of the high divergence SNPs were found on chromosome 12, indicating population specific adaptation.

## 4 Discussion

In this study we showed how low coverage genome sequencing can be used to resolve Atlantic herring population structure. Our population analysis showed that there are most likely four herring populations in Faroese waters corresponding to the management stocks NSSH, NSAH, ISSH and FASH. The results indicate that the local FASH is indeed a distinct biological population and that it is genetically similar to ISSH. This is the first time the FASH population and population mixing in Faroese waters has been investigated in such detail. It was evident that there is more mixing in Faroese waters than previously thought. For example, spawning NSAH individuals have previously not been registered in Faroese fjords during the autumn. This is because the traditional methods are not able to distinguish between FASH and NSAH as the main distinction is based on the catch location. Thus, it is possible that this phenomenon is more common than anticipated (but see also discussion in Section 4.2). This highlighting the need for genetic assignment methods. In addition, we confirmed the findings by Shaw et al. (1999) that ISSH and NSSH can be distinguished from each other using genomic approaches. The selected panel showed promise in assigning herring individuals to population; in particular for assigning individuals to NSSH, NSAH and FASH/ISSH, but was less successful when distinguishing between FASH and ISSH. This indicates that the SNP panel could be used to assign mixed herring catches to populations in the Northeast Atlantic.

### 4.1 The FASH and ISSH populations

The results strongly indicate that the FASH stock is an isolated population from the other populations in Faroese waters. The cluster and STRUCTURE analysis show that four populations is the most parsimonious explanation for the data, corresponding to the management units (Figs. 2 and 3). However, there is close affiliation between the FASH and the ISSH, with low levels of genetic separation (Fig. 4). In addition, the F_ST_ between FASH and ISSH was much lower than for the other population comparisons (Table 2). The assignment of the assessment samples was also relatively poor for the FASH and ISSH individuals, with 27.1% and 48.3% of individuals not being assigned, respectively. The difficulty with these unassigned individuals was for the most part whether they should be assigned to either FASH or ISSH, indicating that the panel is not able to fully distinguish between these two populations. This could partly be caused by the low sample sizes and the fact that the samples included non-spawning individuals. However, the phenomenon of ‘high-grading bias’ should also be considered, i.e. the selection of a small number of the most differentiated markers from a large pool, leading to overly optimistic expectations for assignment of unknown samples based on results from baseline samples alone (Anderson, 2010). Thus, a part of the apparent differentiation may represent selection of loci with a high sampling variance rather than true genetic differences. In addition, the populations are geographically close, and their spawning times could overlap. ISSH are summer-spawners (July) while FASH are early autumn-spawners (August-September) (Jakobsson et al., 1969; Tåning, 1943), providing good conditions for gene flow between the two populations. It is likely that the two stocks are part of a meta-population, with events of colonisation/extinction and/or extensive gene flow. It is also possible that the population separation has occurred too recently for a large number of differences to have evolved. In addition to time since separation, the effective population size, rate of gene flow, and hybrid fitness affect how fast two populations diverge (Dobzhansky and Wright, 1943). Recent genomic studies have shown that Atlantic herring show strong adaption to ecological conditions and spawning time (Han et al., 2020; Martinez Barrio et al., 2016; Pettersson et al., 2019). However, there might not be enough ecological difference between the Faroese and Icelandic waters for selection pressure to have had a large differentiating effect on the two herring populations.

Another factor that could contribute to the low genetic assignment rate for FASH and ISSH could be the sequencing coverage. The individuals used in the baseline were sequenced at low coverage (average 2.6x), which makes the genotypes inferred from these data more uncertain, e.g., sequencing errors are more likely to be incorporated into the dataset as a heterozygous genotype, or only one allele from a heterozygote is sequenced, resulting in a homozygous genotype being called. Theoretically, these uncertainties should be distributed equally throughout the whole data set, provided uniform sequence coverage, which was not the case (Table 1). This uneven coverage makes the called genotypes less certain for the populations with lower coverage and potentially results in higher false homozygous genotypes. This might especially affect the ISSH samples, which had the lowest coverage (1.6x average coverage, compared to 2.4–4.5x). This could be the reason that we detected four clusters when analysing the sequenced individuals, but not when we tried to assign the assessment individuals (i.e. the difference detected between FASH and ISSH is due to uneven sequence coverage and not to true genetic difference). However, the poor DNA quality of the assessment individuals is likely the primary cause of the relatively poor assignment success. On a final note, the uncertainty surrounding the biology of the FASH population also makes it difficult to study. For example, there is not a clear spawning pattern described, with a specific place and time every year. Consequently, it is difficult to obtain sufficiently large samples of locally spawning individuals that would provide an ideal sample for inferring population structure. In addition, there is no regular monitoring of the population, and samples are only collected from local fishing boats when they happen to encounter a school of herring in one of the fjords.

### 4.2 Population mixing

It was evident (Fig. 6) that there is more population mixing in Faroese waters than previously thought. Roughly one third of the FASH assessment individuals were assigned to ISSH using genetic assignment, and one third was assigned to NSAH, while the remaining individuals were assigned to FASH. The 13 spawning FASH individuals that were collected for this study were captured from mid-August to mid-October. The genetic analyses of these individuals revealed that six most likely belonged to the NSAH, three to ISSH and four to FASH. Individuals being assigned to ISSH is likely caused by the limited discriminatory power of the SNP panel to unambiguously distinguish between individuals from the FASH and ISSH populations. This is supported by the assessment samples from Icelandic waters, which seem to contain individuals from the FASH population. This is unexpected as the FASH population is believed to be a local and non-migratory population. An alternative explanation could be that the FASH larva drifted into Icelandic waters where the herring then matured. Further investigations are needed to resolve these observations. Nevertheless, according to these results, there appear to be NSAH individuals spawning in Faroese waters, which is an unexpected finding. NSAH have previously been caught in Faroese waters during the summer in 1990 and 1991 (Jacobsen, 1990, 1991). It has been hypothesised that this migration was aided by a salinity abnormality in the water in the Faroe-Shetland Channel in the early 1990s (Hansen and Kristiansen, 1994). In 2012–2016 another salinity abnormality took place in the North Atlantic (Holliday et al., 2020). All the NSAH individuals that were caught in Faroese waters in autumn for this study, were captured in 2017 and their migration could have been aided by this salinity abnormality. This could also be affected by ongoing climate change rendering conditions for spawning for NSAH more suitable with warming conditions. However, to infer that this is a likely explanation at this stage is highly speculative.

### 4.3 Sample selection and limitations

A major limitation of this study is the use of non-spawning individuals in some of the baseline populations. As previously mentioned, the uncertainties surrounding the FASH exact spawning place and time, made collecting only spawning individuals for the baseline impossible. Therefore, the samples were collected according to the available biological information about the stocks (i.e., their distribution, spawning type and maturity stage in relation to the sampling time and sampling place). The individuals to be used in the baseline were chosen based on traditional assignment (see Section 2.1), to ensure a roughly even representation of the four populations in the sequenced samples. However, the baseline populations were defined later, using genetic clustering (see Section 2.5). Thus, the baseline populations were not based on traditional assignment but on the population analyses. We believe this is the best approach when it is not possible to collect individuals from their spawning grounds.

### 4.4 Ecological adaption

The difference in F_ST_ values when comparing populations using the full data set versus the SNP panel data set (Table 2), confirms that there is low genetic differentiation in most parts of the herring genome, but specific regions show elevated genetic differentiation. When investigating the SNPs with the highest F_ST_, there were several specific genomic regions that showed elevated differentiation among the different populations (Fig. 7). The regions on chromosomes 12, 15 and 19 and a few SNPs on chromosomes 2, 7 and 8, have previously been associated with ecological adaption in Atlantic herring (Han et al., 2020; Pettersson et al., 2019). Among the SNPs that differentiate between the populations, approximately half are located in an area on chromosome 12 corresponding to the supergene defined by Pettersson et al. (2019). This was most evident between NSAH and the other populations.

### 4.5 Uses and implications in industry and monitoring

The correct assignment of individual fish to a population is of high value for maintaining sustainable herring fisheries. The SNPs identified in this study could be very useful for monitoring the herring fisheries and to obtain a realistic picture of the degree of population mixing and ultimately exploitation levels of individual populations. Not being able to distinguish between FASH and ISSH is a challenge, but the FASH fishery is small (around 12,000 t as compared to 650,000 t of NSSH in 2020 (Anon, 2020; ICES, 2020a)) and local to the Faroe Islands. Our SNP panel could be used to assign herring from fisheries to NSAH, NSSH or ISSH/FASH. The assignment success for a combined ISSH and FASH stock (ISSH-FASH) was 88.2%, with 100.0% agreement between genetic and traditional assignment (Supplementary Table A5 in Appendix A). Furthermore, additional tests with larger baseline sample sizes collected at spawning time and with another genotyping platform and/or better DNA quality could reveal improved resolution within the ISSH/FASH complex. Bekkevold et al. (2015) developed a panel with 156 SNPs (not overlapping with the SNPs in this study) for individual assignment to a geographical region (North Atlantic, North Sea, and the British Isles, “Transition” [North Sea–Baltic transition area] and Baltic Sea), with an assignment accuracy of 92% among populations. Our study involved fewer populations, but our SNP panel could assign individuals to one of the three populations (NSAH, NSSH or ISSH/FASH) rather than a geographic region, with similar accuracy and success. This makes our panel particularly useful for dealing with mixed herring catches. Combining both these panels would probably result in an even more powerful tool that could be used to investigate mixed herring catches in most of the North Atlantic Ocean. Furthermore, our SNP panel could be useful for monitoring the herring populations of the North Atlantic. Especially when conducting the annual internationally coordinated herring surveys focusing on the NSSH stock (ICES, 2020b), this would improve the accuracy of the survey indices for NSSH, which are used as input to the stock assessment of NSSH.

## 5 Conclusion

The results from this study show that low coverage sequencing (average 2.6x) can be useful for high gene flow organisms like herring, in order to resolve population structure, but it is important to be aware of uneven coverage. We showed that there are most likely four different herring populations in Faroese waters (corresponding to the management stocks NSSH, NSAH, ISSH and FASH), with low genetic differences between FASH and ISSH. Further studies, with larger sample size, spawning individuals and high-quality DNA are needed to finally confirm whether FASH and ISSH are two distinct biological populations and reveal their relative distribution in Faroese waters. The results are crucial for stock management for example for estimating the actual stock size and exploitation levels, which are important to prevent over-exploitation. Finally, the study has shown that the SNP panel developed in this study could be used in monitoring of herring fisheries as well as in stock management. In concert with SNPs identified in other studies, the SNPs identified here, could be combined into valuable high-resolution tools for assignment of mixed herring catches and thus help to assure sustainable herring fisheries.

## Supplementary Material

Supplementary Figures

Supplementary Tables

## Figures and Tables

**Table 1:** Sequencing coverage of the four Atlantic herring putative populations. Populations are based on traditional methods. NSSH = Norwegian spring-spawning herring, NSAH = North Sea autumn-spawning herring, FASH = Faroese autumnspawning herring, ISSH = Icelandic summer-spawning herring, SD = standard deviation.

Population	No. of individuals	Coverage (x)	Mean coverage ± SD (x)
NSSH	29	74	2.5 ± 0.4
ISSH	30	48	1.6 ± 0.6
NSAH	17	76	4.5 ± 0.8
FASH	27	68	2.5 ± 1.0

**Table 2:** Pairwise population F_ST_ in baseline samples. The full SNP set contained the 4.6 million SNPs from the individual data, while the SNP panel consisted of the 118 selected SNPs. NSSH = Norwegian spring-spawning herring, NSAH = North Sea autumn-spawning herring, FASH = Faroese autumn-spawning herring, ISSH = Icelandic summer-spawning herring.

Population pair	Full SNP set	SNP panel
FASH vs ISSH	0.0007	0.1313
FASH vs NSSH	0.0019	0.4271
ISSH vs NSSH	0.0032	0.4468
FASH vs NSAH	0.0046	0.3495
ISSH vs NSAH	0.0045	0.3815
NSSH vs NSAH	0.0037	0.4357

**Table 3:** Self-assignment of the Atlantic herring baseline populations. Results represent the mean assignment ± standard deviation across 90 tests from Monte-Carlo cross-validation with 50% of individuals used as the training set. Based on the 118 SNP panel. NSSH = Norwegian spring-spawning herring, NSAH = North Sea autumn-spawning herring, FASH = Faroese autumn-spawning herring, ISSH = Icelandic summer-spawning herring.

	FASH	ISSH	NSAH	NSSH
FASH	0.99 ± 0.04	0.01 ± 0.04	0.00 ± 0.00	0.00 ± 0.00
ISSH	0.02 ± 0.04	0.98 ± 0.04	0.00 ± 0.00	0.00 ± 0.00
NSAH	0.00 ± 0.00	0.00 ± 0.00	0.97 ± 0.05	0.03 ± 0.05
NSSH	0.00 ± 0.00	0.00 ± 0.00	0.03 ± 0.03	0.97 ± 0.03

## Data Availability

The sequencing reads are available in the European Nucleotide Archive repository, with accession numbers from ERS4329014 to ERS4329116. Additional data can be obtained by contacting the corresponding author.
