# Supplementary material for: Atlantic herring (Clupea harengus) population structure in the Northeast Atlantic Ocean
Source: Fish Res. Author manuscript; Available in PMC 2023 Feb 15. (PMC7614180; doi:10.1016/j.fishres.2022.106231)
Supplement: Supplementary Figures [file EMS164608-supplement-Supplementary_Figures.docx]

**Appendix B – Supplementary Figures
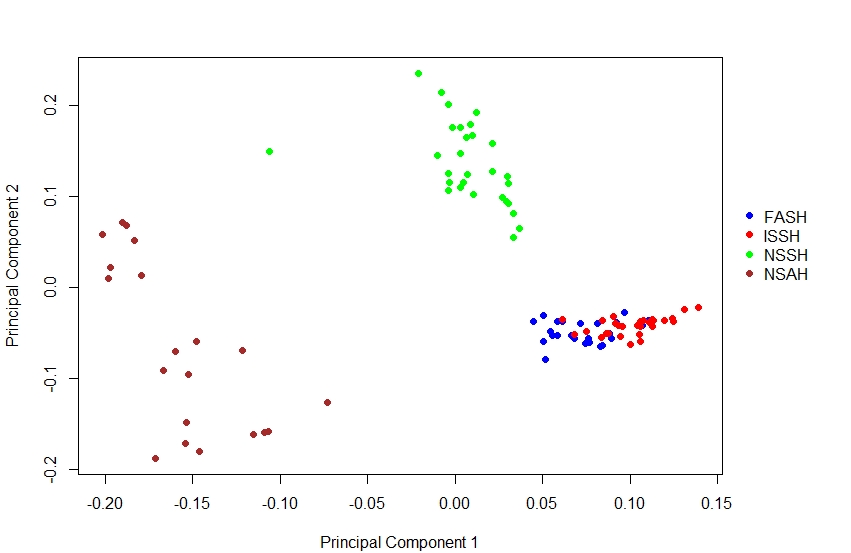
**

**Supplementary Figure A1. Principal component analysis with genotype likelihoods from the full SNP data set (4.9 million SNPs) from the four stocks FASH, ISSH, NSSH, and NSAH.** NSSH = Norwegian spring-spawning herring, NSAH = North Sea autumn-spawning herring, FASH = Faroese autumn spawning herring, and ISSH = Icelandic summer-spawning herring.

| **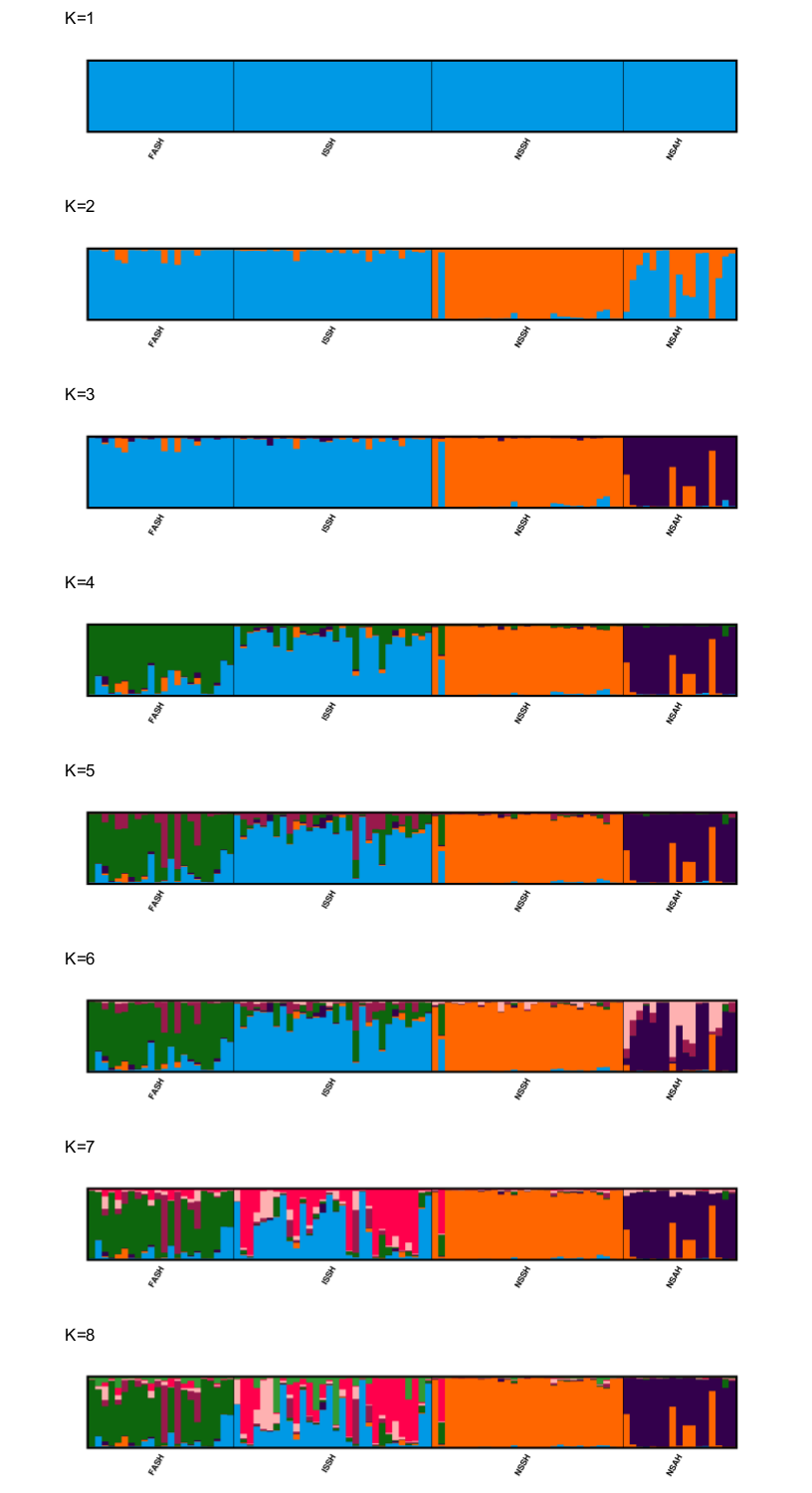** |
| --- |
| **Supplementary Figure A2. Barplots showing the STRUCTURE results for individuals from the NSSH, NSAH, FASH, and ISSH stocks, and K = 1–8.** |
| **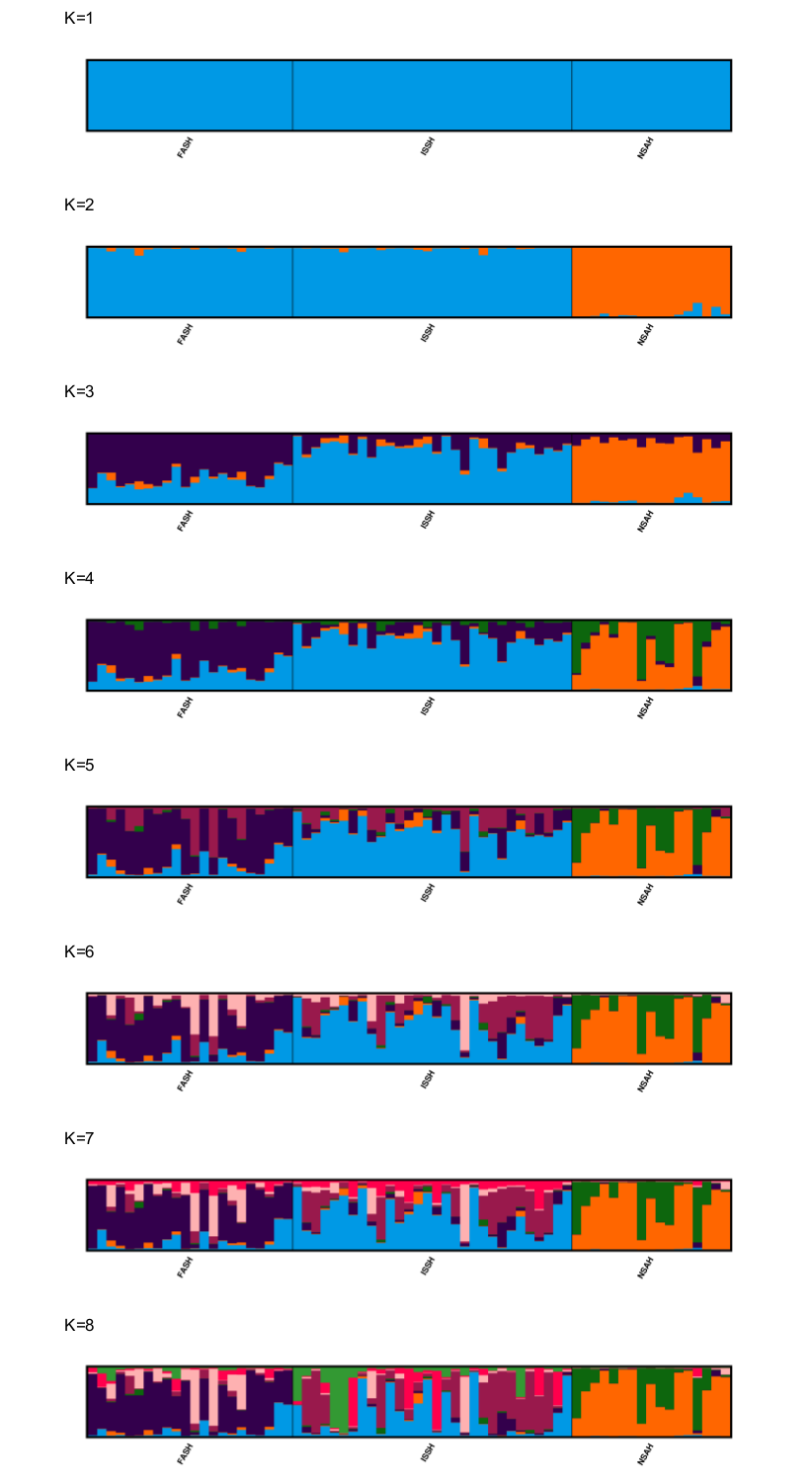** |
| **Supplementary Figure A3. Barplots showing the STRUCTURE results for individuals from the NSAH, FASH, and ISSH stocks, and K = 1–8.** |

| 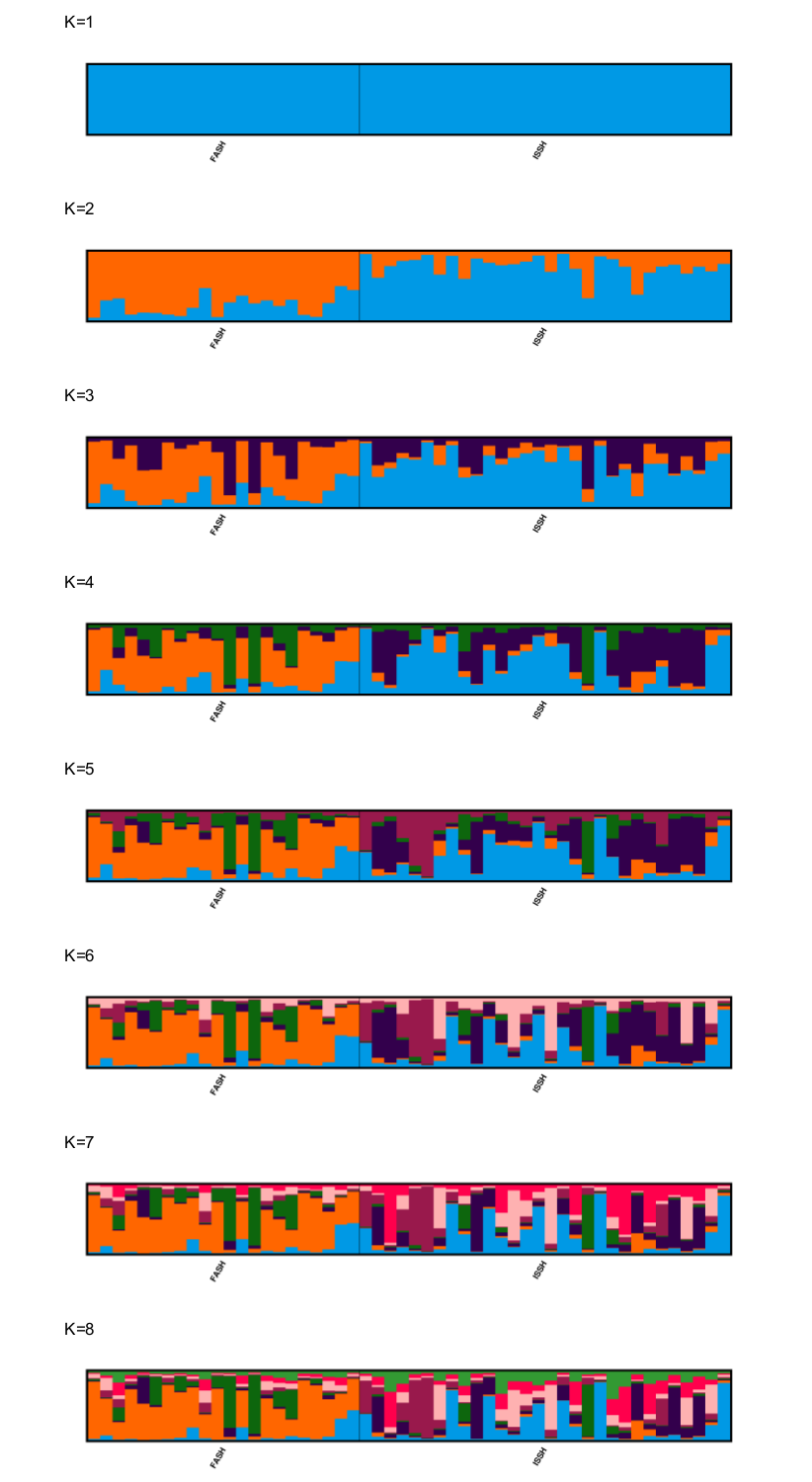 |
| --- |
| **Supplementary Figure A4. Barplots showing the STRUCTURE results for individuals from the FASH and ISSH stocks, and K = 1-8.** |

| **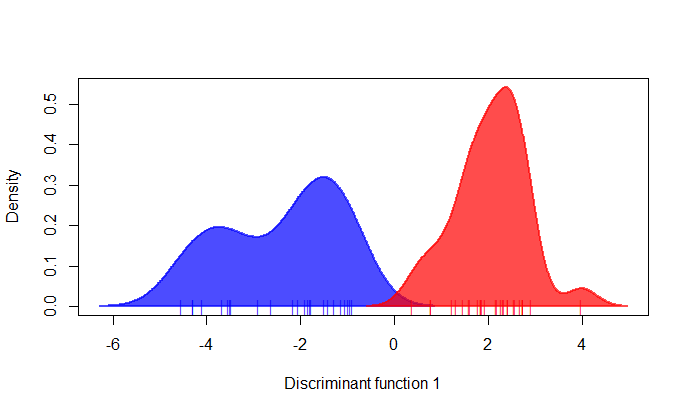** |
| --- |
| **Supplementary Figure A5. DAPC analysis with genotype data from the SNP panel from the FASH and ISSH stocks.** FASH is blue and ISSH is red. |
