# Supplementary material for: Atlantic herring (Clupea harengus) population structure in the Northeast Atlantic Ocean
Source: Fish Res. Author manuscript; Available in PMC 2023 Feb 15. (PMC7614180; doi:10.1016/j.fishres.2022.106231)
Supplement: Supplementary Tables [file EMS164608-supplement-Supplementary_Tables.docx]

**Appendix A – Supplementary Tables**

| **Supplementary Table A1. The maturity stage scale used in this study and the corresponding maturity stage on the WKASMSF 2018 scale.** The Faroese scale is based on Bowers and Holliday (1961). The “Workshop for Advancing Sexual Maturity Staging in Fish” (WKASMSF) 2018 scale has additional stages that are not used in the Faroese scale (ICES, 2018). | | | | |
| --- | --- | --- | --- | --- |
| **Faroese scale** | |  | **WKASMSF 2018 Scale** | |
| **Code** | **Description** |  | **Code** | **Description** |
| 1 | Immature |  | A | Immature |
| 2 | Immature, first time developing |  | A | Immature |
| 3 | Developing, early |  | Bb | Developing and functionally mature |
| 4 | Developing |  | Bb | Developing and functionally mature |
| 5 | Developing, late |  | Bb | Developing and functionally mature |
| 6 | Spawning |  | Ca | Actively spawning |
| 7 | Spent |  | Da | Regressing |
| 8 | Resting |  | Db | Regenerating |

| **Supplementary Table A2. Overview of the Atlantic herring samples used in this study.** The population was inferred from maturity stage (Supplementary Table A1), date and location of the sample (*i.e.,* traditional assignment). NSSH = Norwegian spring-spawning herring, NSAH = North Sea autumn-spawning herring, FASH = Faroese autumn-spawning herring, and ISSH = Icelandic summer-spawning herring. Samples marked with RC in column 12 were caught on a research cruise, whereas samples marked F were caught by fishing boats. | | | | | | | | | | | | |
| --- | --- | --- | --- | --- | --- | --- | --- | --- | --- | --- | --- | --- |
| **Sample** | **Date** | **Sample**  **size** | **Maturity stage** | | | | | | | | **Caught on** | **Population** |
|  |  |  | **1** | **2** | **3** | **4** | **5** | **6** | **7** | **8** |  |  |
| 15520045 | 10-07-15 | 3 |  |  | 2 |  | 1 |  |  |  | RC | NSSH |
| 15520047 | 11-07-15 | 9 |  |  | 9 |  |  |  |  |  | RC | NSSH |
| 15520051 | 11-07-15 | 8 |  |  | 5 | 3 |  |  |  |  | RC | NSSH |
| 15520055 | 12-07-15 | 7 |  |  | 6 |  | 1 |  |  |  | RC | NSSH |
| 15520059 | 13-07-15 | 5 |  |  | 4 | 1 |  |  |  |  | RC | NSSH |
| 15520063 | 13-07-15 | 2 |  |  | 2 |  |  |  |  |  | RC | NSSH |
| 20155056 | 03-12-15 | 14 |  |  | 9 | 5 |  |  |  |  | F | NSSH |
| 20175044 | 25-10-17 | 41 |  |  |  | 41 |  |  |  |  | F | NSSH |
| 20160459 | 02-06-16 | 2 |  |  |  | 1 | 1 |  |  |  | F | FASH |
| 20175014 | 15-02-17 | 16 | 1 | 8 | 7 |  |  |  |  |  | F | FASH |
| 20175015 | 15-02-17 | 9 | 3 | 4 | 2 |  |  |  |  |  | F | FASH |
| 20145054 | 07-12-14 | 7 | 2 | 5 |  |  |  |  |  |  | F | FASH |
| 20155014 | 28-08-15 | 8 |  |  |  |  | 2 | 5 |  | 1 | F | FASH |
| 20175036 | 02-10-17 | 16 |  | 6 | 4 | 3 | 3 |  |  |  | F | FASH |
| 20175037 | 17-08-17 | 12 |  |  | 1 | 2 | 6 | 3 |  |  | F | FASH |
| 20175038 | 18-09-17 | 13 |  | 1 | 3 | 3 | 5 | 1 |  |  | F | FASH |
| 20175060 | 15-10-17 | 4 |  |  |  |  |  | 4 |  |  | F | FASH |
| 20185027 | 25-07-18 | 60 |  | 6 | 16 | 17 | 14 | 2 |  | 5 | F | NSAH |
| 20165079 | 01-11-16 | 17 |  | 4 |  | 1 |  | 5 | 7 |  | F | NSAH |
| 20175020 | 17-02-17 | 22 | 12 | 5 | 5 |  |  |  |  |  | RC | ISSH |
| 20175021 | 17-02-17 | 22 | 7 | 1 | 13 |  |  |  |  | 1 | RC | ISSH |
| 20175022 | 22-02-17 | 23 |  |  | 5 | 1 |  |  |  | 17 | RC | ISSH |
| 20175023 | 22-02-17 | 23 |  |  | 9 | 5 | 2 |  |  | 7 | RC | ISSH |

| **Supplementary Table A3. SNPs included in the SNP panel.** The first two columns show the positions on the ASM96633v1 assembly, while the third and fourth columns show a lift over of these SNPs to the Ch_v2.0.2 assembly. Three SNPs did not get a match on the new assembly (NA). | | | |
| --- | --- | --- | --- |
| **Scaffold** | **Position** | **Chromosome** | **Position** |
| NW_012217742.1 | 291754 | NC_045159.1 | 27986531 |
| NW_012217742.1 | 1571378 | NC_045159.1 | 26894132 |
| NW_012217742.1 | 1645215 | NA | NA |
| NW_012217742.1 | 1750573 | NC_045159.1 | 26808981 |
| NW_012217743.1 | 11138418 | NC_045163.1 | 5207821 |
| NW_012217989.1 | 1112846 | NC_045165.1 | 20962383 |
| NW_012218012.1 | 143803 | NC_045175.1 | 13256839 |
| NW_012218384.1 | 402018 | NC_045159.1 | 30500848 |
| NW_012218579.1 | 25179 | NA | NA |
| NW_012218664.1 | 653693 | NC_045163.1 | 24732915 |
| NW_012218664.1 | 733146 | NC_045163.1 | 24788574 |
| NW_012218664.1 | 1148999 | NC_045163.1 | 25148010 |
| NW_012218741.1 | 1190087 | NC_045171.1 | 2877858 |
| NW_012219336.1 | 1044560 | NC_045157.1 | 24365761 |
| NW_012219336.1 | 5298114 | NC_045157.1 | 28164686 |
| NW_012219445.1 | 96258 | NC_045166.1 | 8922045 |
| NW_012219445.1 | 165218 | NC_045166.1 | 8842154 |
| NW_012219445.1 | 173389 | NC_045166.1 | 8837095 |
| NW_012219445.1 | 175606 | NC_045166.1 | 8834854 |
| NW_012219445.1 | 179453 | NC_045166.1 | 8831013 |
| NW_012219445.1 | 196078 | NC_045166.1 | 8813745 |
| NW_012219460.1 | 898542 | NC_045166.1 | 7750607 |
| NW_012219460.1 | 905136 | NC_045166.1 | 7757217 |
| NW_012219460.1 | 1341630 | NC_045166.1 | 8190209 |
| NW_012219506.1 | 567625 | NC_045159.1 | 20739623 |
| NW_012219682.1 | 4708738 | NC_045162.1 | 8876719 |
| NW_012219682.1 | 4726466 | NC_045162.1 | 8894190 |
| NW_012219847.1 | 8345 | NC_045166.1 | 9027008 |
| NW_012219847.1 | 22108 | NC_045166.1 | 9040907 |
| NW_012219847.1 | 25766 | NC_045166.1 | 9044545 |
| NW_012219847.1 | 67409 | NC_045166.1 | 9076266 |
| NW_012219906.1 | 63409 | NC_045177.1 | 8220147 |
| NW_012220149.1 | 1432941 | NC_045163.1 | 27233575 |
| NW_012220427.1 | 1107464 | NC_045159.1 | 23661064 |
| NW_012220594.1 | 71769 | NC_045159.1 | 29607671 |
| NW_012220594.1 | 170110 | NC_045159.1 | 29797427 |
| NW_012220693.1 | 765733 | NC_045155.1 | 2603215 |
| NW_012220736.1 | 2389822 | NC_045167.1 | 11970281 |
| NW_012220745.1 | 235216 | NC_045152.1 | 167461 |
| NW_012220751.1 | 2658812 | NC_045166.1 | 10985557 |
| NW_012220751.1 | 2665924 | NC_045166.1 | 10978449 |
| NW_012220751.1 | 2673194 | NC_045166.1 | 10971168 |
| NW_012220751.1 | 2678994 | NC_045166.1 | 10965368 |
| NW_012220751.1 | 2682884 | NC_045166.1 | 10961472 |
| NW_012220751.1 | 2684212 | NC_045166.1 | 10959775 |
| NW_012220751.1 | 2687058 | NC_045166.1 | 10956917 |
| NW_012220751.1 | 2754812 | NC_045166.1 | 10894531 |
| NW_012220751.1 | 2757724 | NC_045166.1 | 10891793 |
| NW_012220751.1 | 2761727 | NC_045166.1 | 10887748 |
| NW_012220751.1 | 2813744 | NC_045166.1 | 10834560 |
| NW_012220751.1 | 2841822 | NC_045166.1 | 10806759 |
| NW_012220751.1 | 2841887 | NC_045166.1 | 10806694 |
| NW_012220937.1 | 2110026 | NC_045152.1 | 20681816 |
| NW_012220981.1 | 204107 | NC_045152.1 | 26895549 |
| NW_012221078.1 | 142660 | NC_045157.1 | 14659370 |
| NW_012221132.1 | 1318993 | NC_045177.1 | 5631972 |
| NW_012221139.1 | 85504 | NC_045175.1 | 1012714 |
| NW_012221139.1 | 803980 | NC_045175.1 | 1728695 |
| NW_012221149.1 | 3392388 | NC_045159.1 | 18925480 |
| NW_012221175.1 | 433365 | NC_045154.1 | 31544463 |
| NW_012221193.1 | 1724909 | NC_045176.1 | 8925536 |
| NW_012221369.1 | 559543 | NC_045159.1 | 28753841 |
| NW_012221369.1 | 593471 | NC_045159.1 | 28788736 |
| NW_012221476.1 | 199179 | NC_045159.1 | 29053446 |
| NW_012221476.1 | 302577 | NC_045159.1 | 29163472 |
| NW_012221549.1 | 31624 | NC_045159.1 | 12153938 |
| NW_012221549.1 | 31652 | NC_045159.1 | 12153966 |
| NW_012221562.1 | 165587 | NC_045163.1 | 22512597 |
| NW_012221562.1 | 315068 | NC_045163.1 | 22370317 |
| NW_012221562.1 | 1136530 | NC_045163.1 | 21606820 |
| NW_012221562.1 | 1252026 | NC_045163.1 | 21490121 |
| NW_012221562.1 | 1676685 | NC_045163.1 | 21104841 |
| NW_012221562.1 | 1752836 | NC_045163.1 | 21028891 |
| NW_012221562.1 | 2033187 | NC_045163.1 | 20747876 |
| NW_012221562.1 | 2172494 | NC_045163.1 | 20622482 |
| NW_012221562.1 | 2677025 | NC_045163.1 | 20144416 |
| NW_012221562.1 | 3047253 | NC_045163.1 | 19847301 |
| NW_012221562.1 | 3753761 | NC_045163.1 | 19213992 |
| NW_012221562.1 | 3791106 | NC_045163.1 | 19171991 |
| NW_012221562.1 | 4254530 | NC_045163.1 | 18739179 |
| NW_012221562.1 | 4636916 | NC_045163.1 | 18383181 |
| NW_012221617.1 | 1005462 | NC_045158.1 | 4801784 |
| NW_012221617.1 | 1740524 | NC_045158.1 | 5500145 |
| NW_012221794.1 | 2767118 | NC_045170.1 | 20622288 |
| NW_012221794.1 | 2807432 | NC_045170.1 | 20568801 |
| NW_012221794.1 | 2838904 | NC_045170.1 | 20534628 |
| NW_012221794.1 | 2909663 | NC_045170.1 | 20477940 |
| NW_012221794.1 | 2947918 | NC_045170.1 | 20432914 |
| NW_012221794.1 | 2951673 | NC_045170.1 | 20429342 |
| NW_012221794.1 | 2951756 | NC_045170.1 | 20429259 |
| NW_012221794.1 | 2956058 | NC_045170.1 | 20426127 |
| NW_012221794.1 | 2960191 | NC_045170.1 | 20421878 |
| NW_012222014.1 | 56045 | NC_045159.1 | 23558597 |
| NW_012222014.1 | 59352 | NC_045159.1 | 23555387 |
| NW_012222014.1 | 125637 | NC_045159.1 | 23487528 |
| NW_012222391.1 | 3030933 | NC_045172.1 | 20489001 |
| NW_012222450.1 | 579419 | NC_045153.1 | 19747598 |
| NW_012222613.1 | 762980 | NC_045159.1 | 25920715 |
| NW_012222613.1 | 1968902 | NC_045159.1 | 24767427 |
| NW_012222635.1 | 2142478 | NC_045155.1 | 8095989 |
| NW_012222635.1 | 2451183 | NC_045155.1 | 7882872 |
| NW_012222666.1 | 41390 | NC_045161.1 | 10609417 |
| NW_012222666.1 | 335967 | NC_045164.1 | 22668394 |
| NW_012222967.1 | 5942940 | NA | NA |
| NW_012223336.1 | 438055 | NC_045167.1 | 23566716 |
| NW_012223366.1 | 1991360 | NC_045160.1 | 23097633 |
| NW_012223495.1 | 341453 | NC_045177.1 | 7535262 |
| NW_012223790.1 | 95201 | NC_045163.1 | 22763315 |
| NW_012223790.1 | 233094 | NC_045163.1 | 22974111 |
| NW_012223790.1 | 431581 | NC_045163.1 | 23153337 |
| NW_012223790.1 | 497322 | NC_045163.1 | 23218332 |
| NW_012223790.1 | 498560 | NC_045163.1 | 23219394 |
| NW_012223790.1 | 677950 | NC_045163.1 | 23376172 |
| NW_012223854.1 | 63007 | NC_045175.1 | 2824217 |
| NW_012223947.1 | 1483559 | NC_045172.1 | 11047398 |
| NW_012223953.1 | 645736 | NC_045172.1 | 19282785 |
| NW_012224230.1 | 1053362 | NC_045155.1 | 3453490 |
| NW_012224246.1 | 135489 | NC_045173.1 | 24930554 |

**Supplementary Table A4. The individuals constituting the reference populations defined in a** **discriminant analysis of principal components (DAPC)**. Column 3 indicates the traditional assignment of the individuals, while column 9 shows the reference population that the DAPC analysis revealed. Norwegian spring-spawning herring (NSSH), North Sea autumn-spawning herring (NSAH), Faroese autumn spawning herring (FASH), and Icelandic summer-spawning herring (ISSH). The description of the maturity stages can be seen in Supplementary Table A1. Column 5 indicates weather the otolith nuclei were hyaline (H) or opaque (O). Columns 6, 7, and 8 show the time and location of catch.

| **Sample ID** | **ShortID** | **Stock Traditional** | **Maturity stage** | **Otolith** | **Date** | **Latitude** | **Longitude** | **Reference population** |
| --- | --- | --- | --- | --- | --- | --- | --- | --- |
| 20155014_1 | FASH1 | FASH | 5 | H | 28.08.15 | -7.0 | 62.2 | NSAH |
| 20145054_4 | FASH10 | FASH | 2 | H | 07.12.14 | -6.8 | 62.2 | FASH |
| 20145054_5 | FASH11 | FASH | 2 | H | 07.12.14 | -6.8 | 62.2 | FASH |
| 20175014_14 | FASH12 | FASH | 3 | H | 15.02.17 | -6.6 | 62.3 | FASH |
| 20160459_1 | FASH13 | FASH | 4 | H | 02.06.16 | -7.0 | 62.2 | FASH |
| 20175014_3 | FASH15 | FASH | 2 | H | 15.02.17 | -6.6 | 62.3 | FASH |
| 20175014_4 | FASH16 | FASH | 2 | H | 15.02.17 | -6.6 | 62.3 | FASH |
| 20175014_5 | FASH17 | FASH | 2 | H | 15.02.17 | -6.6 | 62.3 | FASH |
| 20175014_6 | FASH18 | FASH | 2 | H | 15.02.17 | -6.6 | 62.3 | FASH |
| 20175014_7 | FASH19 | FASH | 3 | H | 15.02.17 | -6.6 | 62.3 | FASH |
| 20155014_2 | FASH2 | FASH | 6 | H | 28.08.15 | -7.0 | 62.2 | NSAH |
| 20175014_9 | FASH20 | FASH | 2 | H | 15.02.17 | -6.6 | 62.3 | FASH |
| 20175014_10 | FASH21 | FASH | 1 | H | 15.02.17 | -6.6 | 62.3 | FASH |
| 20175014_11 | FASH22 | FASH | 3 | H | 15.02.17 | -6.6 | 62.3 | FASH |
| 20175015_3 | FASH23 | FASH | 2 | H | 15.02.17 | -6.8 | 62.1 | FASH |
| 20175015_4 | FASH24 | FASH | 2 | H | 15.02.17 | -6.8 | 62.1 | FASH |
| 20175015_5 | FASH25 | FASH | 2 | H | 15.02.17 | -6.8 | 62.1 | FASH |
| 20175015_7 | FASH27 | FASH | 1 | H | 15.02.17 | -6.8 | 62.1 | FASH |
| 20175015_8 | FASH28 | FASH | 1 | H | 15.02.17 | -6.8 | 62.1 | FASH |
| 20175015_9 | FASH29 | FASH | 1 | H | 15.02.17 | -6.8 | 62.1 | FASH |
| 20155014_3 | FASH3 | FASH | 6 | H | 28.08.15 | -7.0 | 62.2 | FASH |
| 20175015_10 | FASH30 | FASH | 3 | H | 15.02.17 | -6.8 | 62.1 | FASH |
| 20155014_5 | FASH4 | FASH | 5 | H | 28.08.15 | -7.0 | 62.2 | NSAH |
| 20155014_7 | FASH6 | FASH | 6 | H | 28.08.15 | -7.0 | 62.2 | NSAH |
| 20155014_9 | FASH7 | FASH | 8 | H | 28.08.15 | -7.0 | 62.2 | FASH |
| 20155014_10 | FASH8 | FASH | 6 | H | 28.08.15 | -7.0 | 62.2 | FASH |
| 20155014_11 | FASH9 | FASH | 6 | H | 28.08.15 | -7.0 | 62.2 | NSAH |
| 20175020_1 | ISSH1 | ISSH | 1 | H | 17.02.17 | -14.4 | 64.1 | ISSH |
| 20175021_9 | ISSH10 | ISSH | 3 | O | 17.02.17 | -14.3 | 64.1 | ISSH |
| 20175021_4 | ISSH11 | ISSH | 8 | H | 17.02.17 | -14.3 | 64.1 | ISSH |
| 20175021_5 | ISSH12 | ISSH | 1 | H | 17.02.17 | -14.3 | 64.1 | ISSH |
| 20175021_6 | ISSH13 | ISSH | 3 | O | 17.02.17 | -14.3 | 64.1 | ISSH |
| 20175021_7 | ISSH14 | ISSH | 1 | H | 17.02.17 | -14.3 | 64.1 | ISSH |
| 20175022_1 | ISSH15 | ISSH | 3 | O | 22.02.17 | -25.1 | 64.7 | ISSH |
| 20175022_2 | ISSH16 | ISSH | 3 | H | 22.02.17 | -25.1 | 64.7 | ISSH |
| 20175022_3 | ISSH17 | ISSH | 3 | H | 22.02.17 | -25.1 | 64.7 | ISSH |
| 20175022_4 | ISSH18 | ISSH | 8 | H | 22.02.17 | -25.1 | 64.7 | ISSH |
| 20175022_5 | ISSH19 | ISSH | 8 | H | 22.02.17 | -25.1 | 64.7 | FASH |
| 20175020_2 | ISSH2 | ISSH | 1 | H | 17.02.17 | -14.4 | 64.1 | ISSH |
| 20175022_6 | ISSH20 | ISSH | 8 | H | 22.02.17 | -25.1 | 64.7 | ISSH |
| 20175022_7 | ISSH21 | ISSH | 8 | H | 22.02.17 | -25.1 | 64.7 | ISSH |
| 20175022_8 | ISSH22 | ISSH | 8 | H | 22.02.17 | -25.1 | 64.7 | ISSH |
| 20175023_1 | ISSH23 | ISSH | 4 | H | 22.02.17 | -24.5 | 64.8 | ISSH |
| 20175023_2 | ISSH24 | ISSH | 8 | H | 22.02.17 | -24.5 | 64.8 | ISSH |
| 20175023_3 | ISSH25 | ISSH | 5 | H | 22.02.17 | -24.5 | 64.8 | ISSH |
| 20175023_4 | ISSH26 | ISSH | 4 | H | 22.02.17 | -24.5 | 64.8 | ISSH |
| 20175023_5 | ISSH27 | ISSH | 8 | H | 22.02.17 | -24.5 | 64.8 | ISSH |
| 20175023_6 | ISSH28 | ISSH | 4 | H | 22.02.17 | -24.5 | 64.8 | ISSH |
| 20175023_7 | ISSH29 | ISSH | 3 | H | 22.02.17 | -24.5 | 64.8 | ISSH |
| 20175020_3 | ISSH3 | ISSH | 1 | H | 17.02.17 | -14.4 | 64.1 | ISSH |
| 20175023_8 | ISSH30 | ISSH | 3 | H | 22.02.17 | -24.5 | 64.8 | ISSH |
| 20175020_4 | ISSH4 | ISSH | 1 | H | 17.02.17 | -14.4 | 64.1 | ISSH |
| 20175020_5 | ISSH5 | ISSH | 2 | H | 17.02.17 | -14.4 | 64.1 | ISSH |
| 20175020_6 | ISSH6 | ISSH | 2 | H | 17.02.17 | -14.4 | 64.1 | ISSH |
| 20175020_7 | ISSH7 | ISSH | 2 | H | 17.02.17 | -14.4 | 64.1 | ISSH |
| 20175021_8 | ISSH8 | ISSH | 3 | O | 17.02.17 | -14.3 | 64.1 | ISSH |
| 20175021_2 | ISSH9 | ISSH | 3 | H | 17.02.17 | -14.3 | 64.1 | ISSH |
| 20165079_10 | NSAH10 | NSAH | 2 | H | 01.11.16 | 1.8 | 59.7 | NSAH |
| 20165079_11 | NSAH11 | NSAH | 6 | H | 01.11.16 | 1.8 | 59.7 | NSAH |
| 20165079_12 | NSAH12 | NSAH | 6 | H | 01.11.16 | 1.8 | 59.7 | NSAH |
| 20165079_13 | NSAH13 | NSAH | 7 | H | 01.11.16 | 1.8 | 59.7 | NSAH |
| 20165079_15 | NSAH15 | NSAH | 2 | H | 01.11.16 | 1.8 | 59.7 | NSAH |
| 20165079_16 | NSAH16 | NSAH | 4 | H | 01.11.16 | 1.8 | 59.7 | NSSH |
| 20165079_18 | NSAH18 | NSAH | 2 | H | 01.11.16 | 1.8 | 59.7 | NSAH |
| 20165079_19 | NSAH19 | NSAH | 7 | H | 01.11.16 | 1.8 | 59.7 | NSAH |
| 20165079_2 | NSAH2 | NSAH | 6 | H | 01.11.16 | 1.8 | 59.7 | NSAH |
| 20165079_20 | NSAH20 | NSAH | 2 | H | 01.11.16 | 1.8 | 59.7 | NSAH |
| 20165079_3 | NSAH3 | NSAH | 6 | H | 01.11.16 | 1.8 | 59.7 | NSAH |
| 20165079_4 | NSAH4 | NSAH | 7 | H | 01.11.16 | 1.8 | 59.7 | NSAH |
| 20165079_5 | NSAH5 | NSAH | 7 | H | 01.11.16 | 1.8 | 59.7 | NSAH |
| 20165079_6 | NSAH6 | NSAH | 7 | H | 01.11.16 | 1.8 | 59.7 | NSAH |
| 20165079_7 | NSAH7 | NSAH | 7 | H | 01.11.16 | 1.8 | 59.7 | NSAH |
| 20165079_8 | NSAH8 | NSAH | 7 | H | 01.11.16 | 1.8 | 59.7 | NSAH |
| 20165079_9 | NSAH9 | NSAH | 6 | H | 01.11.16 | 1.8 | 59.7 | NSSH |
| 15520045_26 | NSSH1 | NSSH | 3 | O | 10.07.15 | -12.0 | 62.5 | NSSH |
| 15520055_24 | NSSH10 | NSSH | 3 | O | 12.07.15 | -1.2 | 64.1 | NSSH |
| 15520055_25 | NSSH11 | NSSH | 3 | O | 12.07.15 | -1.2 | 64.1 | NSSH |
| 15520059_23 | NSSH12 | NSSH | 3 | O | 13.07.15 | -5.6 | 64.1 | NSSH |
| 15520059_24 | NSSH13 | NSSH | 3 | O | 13.07.15 | -5.6 | 64.1 | NSSH |
| 15520063_26 | NSSH14 | NSSH | 3 | O | 13.07.15 | -6.7 | 65.0 | NSSH |
| 15520063_27 | NSSH15 | NSSH | 3 | O | 13.07.15 | -6.7 | 65.0 | NSSH |
| 20155056_2 | NSSH17 | NSSH | 3 | O | 03.12.15 | -4.7 | 63.6 | NSSH |
| 20155056_3 | NSSH18 | NSSH | 3 | O | 03.12.15 | -4.7 | 63.6 | NSSH |
| 20155056_4 | NSSH19 | NSSH | 4 | O | 03.12.15 | -4.7 | 63.6 | NSSH |
| 15520045_23 | NSSH2 | NSSH | 5 | O | 10.07.15 | -12.0 | 62.5 | FASH |
| 20155056_5 | NSSH20 | NSSH | 3 | O | 03.12.15 | -4.7 | 63.6 | NSSH |
| 20155056_6 | NSSH21 | NSSH | 3 | O | 03.12.15 | -4.7 | 63.6 | NSSH |
| 20155056_7 | NSSH22 | NSSH | 4 | O | 03.12.15 | -4.7 | 63.6 | NSSH |
| 20155056_8 | NSSH23 | NSSH | 3 | O | 03.12.15 | -4.7 | 63.6 | NSSH |
| 20155056_9 | NSSH24 | NSSH | 3 | O | 03.12.15 | -4.7 | 63.6 | NSSH |
| 20155056_10 | NSSH25 | NSSH | 3 | O | 03.12.15 | -4.7 | 63.6 | NSSH |
| 20155056_12 | NSSH26 | NSSH | 4 | O | 03.12.15 | -4.7 | 63.6 | NSSH |
| 20155056_13 | NSSH27 | NSSH | 3 | O | 03.12.15 | -4.7 | 63.6 | NSSH |
| 20155056_22 | NSSH28 | NSSH | 4 | O | 03.12.15 | -4.7 | 63.6 | NSSH |
| 20155056_23 | NSSH29 | NSSH | 3 | O | 03.12.15 | -4.7 | 63.6 | NSSH |
| 15520045_24 | NSSH3 | NSSH | 3 | O | 10.07.15 | -12.0 | 62.5 | NSSH |
| 20155056_24 | NSSH30 | NSSH | 4 | O | 03.12.15 | -4.7 | 63.6 | NSSH |
| 15520047_22 | NSSH4 | NSSH | 3 | O | 11.07.15 | -8.8 | 63.3 | NSSH |
| 15520047_23 | NSSH5 | NSSH | 3 | O | 11.07.15 | -8.8 | 63.3 | NSSH |
| 15520047_24 | NSSH6 | NSSH | 3 | O | 11.07.15 | -8.8 | 63.3 | NSSH |
| 15520051_23 | NSSH7 | NSSH | 3 | O | 11.07.15 | -4.7 | 63.3 | NSSH |
| 15520051_24 | NSSH8 | NSSH | 3 | O | 11.07.15 | -4.7 | 63.3 | NSSH |
| 15520051_25 | NSSH9 | NSSH | 3 | O | 11.07.15 | -4.7 | 63.3 | NSSH |

**Supplementary Table A5. List of individuals and their assignment using traditional assignment method (column 3) and three different genetic methods (columns 4-6).** The letter b indicates technical replicas.

| **Sample-ID** | **Short ID** | **Traditional** | **AssignPOP** | **GeneClass** | **Adegenet** |
| --- | --- | --- | --- | --- | --- |
| 20145054-1 | FGT1 | FASH | FASH | FASH | ISSH |
| 20175014-18 | FGT10 | FASH | ISSH | ISSH | ISSH |
| 20175014-18 | FGT10b | FASH | ISSH | ISSH | ISSH |
| 20175014-19 | FGT11 | FASH | ISSH | ISSH | ISSH |
| 20175014-19 | FGT11b | FASH | ISSH | ISSH | ISSH |
| 20175014-20 | FGT12 | FASH | FASH | FASH | FASH |
| 20175015-1 | FGT13 | FASH | ISSH | ISSH | ISSH |
| 20175015-2 | FGT14 | FASH | ISSH | ISSH | ISSH |
| 20175036-3 | FGT15 | FASH | NSSH | NSSH | NSSH |
| 20175036-4 | FGT16 | FASH | NSAH | NSAH | NSAH |
| 20175036-4 | FGT16b | FASH | NSAH | NSAH | NSAH |
| 20175036-5 | FGT17 | FASH | FASH | FASH | FASH |
| 20175036-5 | FGT17b | FASH | FASH | FASH | ISSH |
| 20175036-6 | FGT18 | FASH | NSAH | NSAH | NSAH |
| 20175036-6 | FGT18b | FASH | NSAH | NSAH | NSAH |
| 20175036-7 | FGT19 | FASH | ISSH | ISSH | ISSH |
| 20175036-8 | FGT20 | FASH | FASH | FASH | FASH |
| 20175036-9 | FGT21 | FASH | FASH | FASH | FASH |
| 20175036-10 | FGT22 | FASH | FASH | FASH | ISSH |
| 20175036-12 | FGT23 | FASH | ISSH | ISSH | ISSH |
| 20175036-13 | FGT24 | FASH | FASH | FASH | FASH |
| 20175036-15 | FGT25 | FASH | FASH | FASH | FASH |
| 20175036-16 | FGT26 | FASH | NSAH | NSAH | NSAH |
| 20175036-17 | FGT27 | FASH | ISSH | ISSH | ISSH |
| 20175036-18 | FGT28 | FASH | FASH | FASH | ISSH |
| 20175036-19 | FGT29 | FASH | ISSH | FASH | ISSH |
| 20145054-3 | FGT3 | FASH | NSAH | NSAH | NSAH |
| 20175036-20 | FGT30 | FASH | NSAH | NSAH | NSAH |
| 20175037-1 | FGT31 | FASH | ISSH | FASH | ISSH |
| 20175037-2 | FGT32 | FASH | FASH | FASH | FASH |
| 20175037-3 | FGT33 | FASH | ISSH | ISSH | ISSH |
| 20175037-4 | FGT34 | FASH | ISSH | ISSH | ISSH |
| 20175037-5 | FGT35 | FASH | FASH | FASH | FASH |
| 20175037-6 | FGT36 | FASH | FASH | ISSH | ISSH |
| 20175037-10 | FGT37 | FASH | FASH | FASH | ISSH |
| 20175037-12 | FGT38 | FASH | ISSH | ISSH | ISSH |
| 20175037-13 | FGT39 | FASH | FASH | FASH | ISSH |
| 20145054-6 | FGT4 | FASH | ISSH | ISSH | ISSH |
| 20175037-14 | FGT40 | FASH | FASH | FASH | FASH |
| 20175037-15 | FGT41 | FASH | ISSH | ISSH | ISSH |
| 20175037-17 | FGT42 | FASH | FASH | FASH | FASH |
| 20175037-18 | FGT43 | FASH | FASH | ISSH | FASH |
| 20175038-2 | FGT44 | FASH | NSAH | NSAH | NSAH |
| 20175038-3 | FGT45 | FASH | FASH | FASH | ISSH |
| 20175038-5 | FGT46 | FASH | NSAH | NSAH | NSAH |
| 20175038-6 | FGT47 | FASH | FASH | FASH | ISSH |
| 20175038-7 | FGT48 | FASH | NSAH | NSAH | NSAH |
| 20175038-10 | FGT49 | FASH | FASH | FASH | FASH |
| 20145054-7 | FGT5 | FASH | NSSH | NSSH | NSSH |
| 20175038-11 | FGT50 | FASH | FASH | NSAH | ISSH |
| 20175038-12 | FGT51 | FASH | FASH | ISSH | ISSH |
| 20175038-13 | FGT52 | FASH | FASH | ISSH | ISSH |
| 20175038-16 | FGT53 | FASH | FASH | ISSH | ISSH |
| 20175038-17 | FGT54 | FASH | FASH | FASH | FASH |
| 20175038-18 | FGT55 | FASH | ISSH | ISSH | FASH |
| 20175038-19 | FGT56 | FASH | FASH | FASH | FASH |
| 20175060-18 | FGT57 | FASH | ISSH | ISSH | ISSH |
| 20175060-18 | FGT57b | FASH | ISSH | ISSH | ISSH |
| 20175060-21 | FGT58 | FASH | NSAH | NSAH | NSAH |
| 20175060-21 | FGT58b | FASH | NSAH | NSAH | NSAH |
| 20175060-32 | FGT59 | FASH | ISSH | ISSH | ISSH |
| 20175060-32 | FGT59b | FASH | ISSH | ISSH | ISSH |
| 20175014-1 | FGT6 | FASH | FASH | FASH | FASH |
| 20175060-35 | FGT60 | FASH | NSAH | NSAH | NSAH |
| 20175014-12 | FGT7 | FASH | FASH | FASH | FASH |
| 20175014-12 | FGT7b | FASH | FASH | FASH | FASH |
| 20175014-15 | FGT8 | FASH | FASH | FASH | FASH |
| 20175014-15 | FGT8b | FASH | FASH | FASH | FASH |
| 20175014-17 | FGT9 | FASH | FASH | FASH | FASH |
| 20175014-17 | FGT9b | FASH | FASH | FASH | FASH |
| 2085027-1 | HGT1 | NSAH | NSAH | NSAH | NSAH |
| 2085027-10 | HGT10 | NSAH | NSAH | NSAH | NSAH |
| 2085027-11 | HGT11 | NSAH | FASH | FASH | FASH |
| 2085027-13 | HGT12 | NSAH | NSAH | NSAH | NSAH |
| 2085027-15 | HGT13 | NSAH | NSAH | NSAH | NSAH |
| 2085027-16 | HGT14 | NSAH | NSAH | NSAH | NSAH |
| 2085027-17 | HGT15 | NSAH | NSAH | NSAH | NSAH |
| 2085027-18 | HGT16 | NSAH | NSAH | NSAH | NSAH |
| 2085027-19 | HGT17 | NSAH | NSAH | NSAH | NSAH |
| 2085027-20 | HGT18 | NSAH | NSAH | NSAH | NSAH |
| 2085027-21 | HGT19 | NSAH | NSAH | NSAH | NSAH |
| 2085027-2 | HGT2 | NSAH | NSAH | NSAH | NSAH |
| 2085027-22 | HGT20 | NSAH | NSAH | NSAH | NSAH |
| 2085027-23 | HGT21 | NSAH | NSAH | NSAH | NSAH |
| 2085027-24 | HGT22 | NSAH | NSAH | NSAH | NSAH |
| 2085027-25 | HGT23 | NSAH | NSAH | NSAH | NSAH |
| 2085027-26 | HGT24 | NSAH | NSAH | NSAH | NSAH |
| 2085027-27 | HGT25 | NSAH | NSAH | NSAH | NSAH |
| 2085027-28 | HGT26 | NSAH | NSAH | NSAH | NSAH |
| 2085027-29 | HGT27 | NSAH | NSAH | NSAH | NSAH |
| 2085027-30 | HGT28 | NSAH | NSAH | NSAH | NSAH |
| 2085027-33 | HGT29 | NSAH | NSAH | NSAH | NSAH |
| 2085027-34 | HGT30 | NSAH | NSAH | NSAH | NSAH |
| 2085027-36 | HGT31 | NSAH | NSAH | NSAH | NSAH |
| 2085027-37 | HGT32 | NSAH | NSAH | NSAH | NSAH |
| 2085027-38 | HGT33 | NSAH | NSSH | NSSH | NSSH |
| 2085027-39 | HGT34 | NSAH | NSAH | NSAH | NSAH |
| 2085027-40 | HGT35 | NSAH | NSAH | NSAH | NSAH |
| 2085027-41 | HGT36 | NSAH | NSAH | NSAH | NSAH |
| 2085027-42 | HGT37 | NSAH | NSAH | NSAH | NSAH |
| 2085027-43 | HGT38 | NSAH | NSAH | NSAH | NSAH |
| 2085027-44 | HGT39 | NSAH | NSAH | NSAH | NSAH |
| 2085027-4 | HGT4 | NSAH | NSAH | NSAH | NSAH |
| 2085027-45 | HGT40 | NSAH | NSAH | NSAH | NSAH |
| 2085027-46 | HGT41 | NSAH | NSAH | NSAH | NSAH |
| 2085027-48 | HGT42 | NSAH | NSAH | NSAH | NSAH |
| 2085027-49 | HGT43 | NSAH | NSAH | NSAH | NSAH |
| 2085027-50 | HGT44 | NSAH | NSAH | NSAH | NSAH |
| 2085027-54 | HGT45 | NSAH | NSAH | NSAH | NSAH |
| 2085027-55 | HGT46 | NSAH | NSAH | NSAH | NSAH |
| 2085027-56 | HGT47 | NSAH | NSAH | NSAH | NSAH |
| 2085027-57 | HGT48 | NSAH | NSAH | NSAH | NSAH |
| 2085027-58 | HGT49 | NSAH | NSAH | NSAH | NSAH |
| 2085027-5 | HGT5 | NSAH | NSAH | NSAH | NSAH |
| 2085027-59 | HGT50 | NSAH | NSAH | NSAH | NSAH |
| 2085027-60 | HGT51 | NSAH | NSAH | NSAH | NSAH |
| 2085027-62 | HGT52 | NSAH | NSAH | NSAH | NSAH |
| 2085027-63 | HGT53 | NSAH | NSAH | NSAH | NSAH |
| 2085027-64 | HGT54 | NSAH | NSAH | NSAH | NSAH |
| 2085027-65 | HGT55 | NSAH | NSAH | NSAH | NSAH |
| 2085027-66 | HGT56 | NSAH | NSAH | NSAH | NSAH |
| 2085027-67 | HGT57 | NSAH | NSAH | NSAH | NSAH |
| 2085027-68 | HGT58 | NSAH | NSAH | NSAH | NSAH |
| 2085027-69 | HGT59 | NSAH | NSAH | NSAH | NSAH |
| 2085027-6 | HGT6 | NSAH | NSAH | NSAH | NSAH |
| 2085027-70 | HGT60 | NSAH | NSAH | NSAH | NSAH |
| 2085027-7 | HGT7 | NSAH | NSAH | NSAH | NSAH |
| 2085027-8 | HGT8 | NSAH | NSAH | NSAH | NSAH |
| 2085027-9 | HGT9 | NSAH | NSAH | NSAH | NSAH |
| 20175020-9 | IGT1 | ISSH | FASH | FASH | ISSH |
| 20175020-18 | IGT10 | ISSH | ISSH | ISSH | ISSH |
| 20175020-19 | IGT11 | ISSH | FASH | FASH | FASH |
| 20175020-20 | IGT12 | ISSH | ISSH | ISSH | ISSH |
| 20175020-21 | IGT13 | ISSH | FASH | ISSH | ISSH |
| 20175020-22 | IGT14 | ISSH | FASH | FASH | FASH |
| 20175020-23 | IGT15 | ISSH | ISSH | FASH | ISSH |
| 20175021-11 | IGT16 | ISSH | FASH | FASH | FASH |
| 20175021-12 | IGT17 | ISSH | ISSH | ISSH | ISSH |
| 20175021-13 | IGT18 | ISSH | FASH | FASH | ISSH |
| 20175021-13 | IGT18b | ISSH | ISSH | ISSH | ISSH |
| 20175021-14 | IGT19 | ISSH | FASH | FASH | FASH |
| 20175021-14 | IGT19b | ISSH | FASH | FASH | FASH |
| 20175020-10 | IGT2 | ISSH | FASH | FASH | ISSH |
| 20175021-15 | IGT20 | ISSH | ISSH | ISSH | ISSH |
| 20175021-16 | IGT21 | ISSH | FASH | FASH | FASH |
| 20175021-17 | IGT22 | ISSH | FASH | ISSH | ISSH |
| 20175021-18 | IGT23 | ISSH | FASH | FASH | ISSH |
| 20175021-19 | IGT24 | ISSH | FASH | FASH | FASH |
| 20175021-20 | IGT25 | ISSH | ISSH | ISSH | ISSH |
| 20175021-21 | IGT26 | ISSH | ISSH | FASH | ISSH |
| 20175021-22 | IGT27 | ISSH | ISSH | ISSH | ISSH |
| 20175021-23 | IGT28 | ISSH | FASH | FASH | FASH |
| 20175021-24 | IGT29 | ISSH | FASH | FASH | FASH |
| 20175020-11 | IGT3 | ISSH | ISSH | ISSH | FASH |
| 20175020-11 | IGT3b | ISSH | ISSH | ISSH | FASH |
| 20175021-25 | IGT30 | ISSH | ISSH | ISSH | FASH |
| 20175022-11 | IGT31 | ISSH | FASH | ISSH | ISSH |
| 20175022-12 | IGT32 | ISSH | ISSH | FASH | ISSH |
| 20175022-13 | IGT33 | ISSH | FASH | ISSH | ISSH |
| 20175022-14 | IGT34 | ISSH | FASH | FASH | ISSH |
| 20175022-15 | IGT35 | ISSH | FASH | FASH | FASH |
| 20175022-16 | IGT36 | ISSH | FASH | FASH | FASH |
| 20175022-17 | IGT37 | ISSH | FASH | FASH | ISSH |
| 20175022-18 | IGT38 | ISSH | ISSH | ISSH | ISSH |
| 20175022-19 | IGT39 | ISSH | FASH | FASH | FASH |
| 20175020-12 | IGT4 | ISSH | ISSH | FASH | ISSH |
| 20175022-20 | IGT40 | ISSH | FASH | FASH | FASH |
| 20175022-21 | IGT41 | ISSH | ISSH | FASH | ISSH |
| 20175022-22 | IGT42 | ISSH | ISSH | ISSH | ISSH |
| 20175022-23 | IGT43 | ISSH | ISSH | ISSH | ISSH |
| 20175022-24 | IGT44 | ISSH | FASH | FASH | ISSH |
| 20175022-25 | IGT45 | ISSH | ISSH | FASH | ISSH |
| 20175023-11 | IGT46 | ISSH | FASH | FASH | FASH |
| 20175023-12 | IGT47 | ISSH | FASH | FASH | ISSH |
| 20175023-12 | IGT47b | ISSH | FASH | FASH | ISSH |
| 20175023-13 | IGT48 | ISSH | ISSH | ISSH | ISSH |
| 20175023-13 | IGT48b | ISSH | ISSH | ISSH | ISSH |
| 20175023-14 | IGT49 | ISSH | ISSH | ISSH | ISSH |
| 20175023-14 | IGT49b | ISSH | ISSH | ISSH | ISSH |
| 20175020-13 | IGT5 | ISSH | ISSH | ISSH | ISSH |
| 20175023-15 | IGT50 | ISSH | ISSH | ISSH | ISSH |
| 20175023-15 | IGT50b | ISSH | ISSH | ISSH | ISSH |
| 20175023-16 | IGT51 | ISSH | NSSH | NSSH | ISSH |
| 20175023-17 | IGT52 | ISSH | ISSH | FASH | ISSH |
| 20175023-18 | IGT53 | ISSH | FASH | FASH | FASH |
| 20175023-19 | IGT54 | ISSH | ISSH | ISSH | ISSH |
| 20175023-20 | IGT55 | ISSH | FASH | FASH | FASH |
| 20175023-21 | IGT56 | ISSH | FASH | FASH | ISSH |
| 20175023-22 | IGT57 | ISSH | ISSH | ISSH | ISSH |
| 20175023-23 | IGT58 | ISSH | FASH | FASH | ISSH |
| 20175023-24 | IGT59 | ISSH | FASH | FASH | ISSH |
| 20175020-14 | IGT6 | ISSH | FASH | FASH | ISSH |
| 20175023-25 | IGT60 | ISSH | FASH | ISSH | ISSH |
| 20175020-15 | IGT7 | ISSH | FASH | FASH | FASH |
| 20175020-16 | IGT8 | ISSH | ISSH | ISSH | FASH |
| 20175020-17 | IGT9 | ISSH | FASH | ISSH | FASH |
| 15520047-25 | NGT1 | NSSH | NSSH | NSSH | NSSH |
| 15520051-30 | NGT10 | NSSH | NSSH | NSSH | NSSH |
| 15520051-32 | NGT11 | NSSH | NSSH | NSSH | NSSH |
| 15520055-22 | NGT12 | NSSH | FASH | FASH | FASH |
| 15520055-22 | NGT12b | NSSH | ISSH | ISSH | ISSH |
| 15520055-174 | NGT13 | NSSH | FASH | FASH | FASH |
| 15520055-174 | NGT13b | NSSH | FASH | FASH | FASH |
| 15520055-179 | NGT14 | NSSH | NSSH | NSSH | NSSH |
| 15520055-179 | NGT14b | NSSH | NSSH | NSSH | NSSH |
| 15520055-180 | NGT15 | NSSH | NSSH | NSSH | NSSH |
| 15520055-180 | NGT15b | NSSH | NSSH | NSSH | NSSH |
| 15520055-181 | NGT16 | NSSH | FASH | ISSH | NSSH |
| 15520059-25 | NGT17 | NSSH | NSSH | NSSH | NSSH |
| 15520059-26 | NGT18 | NSSH | NSSH | NSSH | NSSH |
| 15520059-27 | NGT19 | NSSH | NSSH | NSSH | NSSH |
| 15520047-26 | NGT2 | NSSH | NSSH | NSSH | NSSH |
| 15520047-26 | NGT2b | NSSH | NSSH | NSSH | NSSH |
| 20175044-1 | NGT20 | NSSH | NSSH | NSSH | NSSH |
| 20175044-2 | NGT21 | NSSH | NSSH | NSSH | NSSH |
| 20175044-3 | NGT22 | NSSH | NSSH | NSSH | NSSH |
| 20175044-4 | NGT23 | NSSH | NSSH | NSSH | NSSH |
| 20175044-5 | NGT24 | NSSH | NSSH | NSSH | NSSH |
| 20175044-5 | NGT24b | NSSH | NSSH | NSSH | NSSH |
| 20175044-8 | NGT25 | NSSH | NSSH | NSSH | NSSH |
| 20175044-8 | NGT25b | NSSH | NSSH | NSSH | NSSH |
| 20175044-10 | NGT26 | NSSH | NSSH | NSSH | NSSH |
| 20175044-10 | NGT26b | NSSH | NSSH | NSSH | NSSH |
| 20175044-11 | NGT27 | NSSH | NSSH | NSSH | NSSH |
| 20175044-11 | NGT27b | NSSH | NSSH | NSSH | NSSH |
| 20175044-12 | NGT28 | NSSH | NSSH | NSSH | NSSH |
| 20175044-14 | NGT29 | NSSH | NSSH | NSSH | NSSH |
| 15520047-27 | NGT3 | NSSH | NSSH | NSSH | NSSH |
| 15520047-27 | NGT3b | NSSH | NSSH | NSSH | NSSH |
| 20175044-16 | NGT30 | NSSH | NSSH | NSSH | NSSH |
| 20175044-17 | NGT31 | NSSH | NSSH | NSSH | NSSH |
| 20175044-18 | NGT32 | NSSH | NSSH | NSSH | NSSH |
| 20175044-19 | NGT33 | NSSH | NSSH | NSSH | NSSH |
| 20175044-20 | NGT34 | NSSH | NSSH | NSSH | NSSH |
| 20175044-21 | NGT35 | NSSH | NSSH | NSSH | NSSH |
| 20175044-22 | NGT36 | NSSH | NSSH | NSSH | NSSH |
| 20175044-23 | NGT37 | NSSH | NSSH | NSSH | NSSH |
| 20175044-24 | NGT38 | NSSH | NSSH | NSSH | NSSH |
| 20175044-25 | NGT39 | NSSH | FASH | FASH | FASH |
| 15520047-28 | NGT4 | NSSH | NSSH | NSSH | NSSH |
| 20175044-28 | NGT40 | NSSH | NSSH | NSSH | NSSH |
| 20175044-29 | NGT41 | NSSH | NSSH | NSSH | NSSH |
| 20175044-31 | NGT42 | NSSH | NSSH | NSSH | NSSH |
| 20175044-32 | NGT43 | NSSH | NSSH | NSSH | NSSH |
| 20175044-33 | NGT44 | NSSH | NSSH | NSSH | NSSH |
| 20175044-34 | NGT45 | NSSH | NSSH | NSSH | NSSH |
| 20175044-35 | NGT46 | NSSH | NSSH | NSSH | NSSH |
| 20175044-36 | NGT47 | NSSH | NSSH | NSSH | NSSH |
| 20175044-37 | NGT48 | NSSH | NSSH | NSSH | NSSH |
| 20175044-38 | NGT49 | NSSH | NSSH | NSSH | NSSH |
| 15520047-29 | NGT5 | NSSH | NSSH | NSSH | NSSH |
| 20175044-39 | NGT50 | NSSH | NSSH | NSSH | NSSH |
| 20175044-40 | NGT51 | NSSH | NSSH | NSSH | NSSH |
| 20175044-41 | NGT52 | NSSH | NSSH | NSSH | NSSH |
| 20175044-42 | NGT53 | NSSH | NSSH | NSSH | NSSH |
| 20175044-43 | NGT54 | NSSH | NSSH | NSSH | NSSH |
| 20175044-44 | NGT55 | NSSH | NSSH | NSSH | NSSH |
| 20175044-45 | NGT56 | NSSH | NSSH | NSSH | NSSH |
| 20175044-46 | NGT57 | NSSH | NSSH | NSSH | NSSH |
| 20175044-47 | NGT58 | NSSH | NSSH | NSSH | NSSH |
| 20175044-48 | NGT59 | NSSH | NSSH | NSSH | NSSH |
| 15520047-30 | NGT6 | NSSH | NSSH | NSSH | NSSH |
| 20175044-49 | NGT60 | NSSH | NSSH | NSSH | NSSH |
| 15520051-27 | NGT7 | NSSH | NSSH | NSSH | NSSH |
| 15520051-28 | NGT8 | NSSH | NSSH | NSSH | NSSH |
| 15520051-29 | NGT9 | NSSH | NSSH | NSSH | NSSH |

| **Supplementary Table A6. Summary of assignment results and comparison between genetic and traditional methods of assignment.** In column 3 ‘Assigned’ refers to the number of individuals where all three genetic algorithms assign to the same baseline populations. In column 5 ‘Agreement’ refers to the number of samples where the genetic assignment and traditional population identification methods agree on the population of origin. | | | | | |
| --- | --- | --- | --- | --- | --- |
|  | **Number of**  **samples** | **Assigned** | **% Assigned** | **Agreement** | **% Agreement** |
| **FASH** | 59 | 43 | 72.9 | 17 | 39.5 |
| **ISSH** | 60 | 31 | 51.7 | 15 | 48.4 |
| **NSSH** | 60 | 59 | 98.3 | 56 | 94.9 |
| **NSAH** | 59 | 59 | 100.0 | 57 | 96.6 |
| **Total** | 238 | 192 | 80.7 | 145 | 75.5 |
